# Xylo-Oligosaccharide Utilization by Engineered *Saccharomyces cerevisiae* to Produce Ethanol

**DOI:** 10.3389/fbioe.2022.825981

**Published:** 2022-02-15

**Authors:** Dielle Pierotti Procópio, Emanuele Kendrick, Rosana Goldbeck, André Ricardo de Lima Damasio, Telma Teixeira Franco, David J. Leak, Yong-Su Jin, Thiago Olitta Basso

**Affiliations:** ^1^ Department of Chemical Engineering, Escola Politécnica, University of São Paulo, São Paulo, Brazil; ^2^ Department of Biology and Biochemistry, Faculty of Sciences, University of Bath, Bath, United Kingdom; ^3^ School of Food Engineering, University of Campinas, Campinas, Brazil; ^4^ Department of Biochemistry and Tissue Biology, Institute of Biology, University of Campinas, Campinas, Brazil; ^5^ Interdisciplinary Center of Energy Planning, University of Campinas, Campinas, Brazil; ^6^ School of Chemical Engineering, University of Campinas, Campinas, Brazil; ^7^ DOE Center for Advanced Bioenergy and Bioproducts Innovation, University of Illinois at Urbana-Champaign, Urbana, IL, United States; ^8^ Department of Food Science and Nutrition, University of Illinois at Urbana-Champaign, Urbana, IL, United States

**Keywords:** *Saccharomyces cerevisiae*, xylo-oligosaccharides, lignocellulosic ethanol, xylanases, xylose

## Abstract

The engineering of xylo-oligosaccharide-consuming *Saccharomyces cerevisiae* strains is a promising approach for more effective utilization of lignocellulosic biomass and the development of economic industrial fermentation processes. Extending the sugar consumption range without catabolite repression by including the metabolism of oligomers instead of only monomers would significantly improve second-generation ethanol production This review focuses on different aspects of the action mechanisms of xylan-degrading enzymes from bacteria and fungi, and their insertion in *S. cerevisiae* strains to obtain microbial cell factories able of consume these complex sugars and convert them to ethanol. Emphasis is given to different strategies for ethanol production from both extracellular and intracellular xylo-oligosaccharide utilization by *S. cerevisiae* strains. The suitability of *S. cerevisiae* for ethanol production combined with its genetic tractability indicates that it can play an important role in xylan bioconversion through the heterologous expression of xylanases from other microorganisms.

## Background

High fermentative capacity and robustness make *Saccharomyces cerevisiae* the microorganism of choice for ethanol production. *S. cerevisiae* strains are the most widely used in the ethanol industry with a well-characterized genome sequence, besides being an efficient expression system for recombinant enzyme production (Hou et al., 2012; Fang et al., 2017; Oh and Jin, 2020). Industrial *S. cerevisiae* strains are highly tolerant against various stresses in industrial environments, such as low pH, high osmotic pressure, high alcohol concentration, and phage contamination. In addition, this yeast presents high ethanol productivity, resulting from the naturally selective process that occurs during the successive fermentations involving pitching between fermentation tanks (Della-Bianca et al., 2014; Nielsen, 2019).

Economic production of second-generation biofuels should include the efficient and simultaneous co-fermentation of all hydrolysable sugars derived from cellulose and hemicellulose (Li et al., 2015). Lignocellulose-derived ethanol provides environmental and economic benefits, as significant reductions in the disposal of solid wastes, and less air pollution, besides representing a promising industry in the expected transition from fossil to renewable fuels and chemicals. This biofuel is an environmentally friendly liquid fuel because the exhaust carbon dioxide is being taken up by growing plant biomass, contributing to the reduction of CO_2_ content in the air, which in turn, contributes to the equilibrium of the earth’s atmosphere (Palmqvist and Hahn-Hägerdal, 2000a; Jacobsen and Wyman, 2000; Galbe and Zacchi, 2002).

Hemicellulose and cellulose are the major components of the secondary layers of the cell wall in wood fiber, shaping the well-known natural composition of, lignocellulosic biomass, along with lignin and minor components such as extractives and minerals (Gírio et al., 2010). Lignocellulose represents the most abundant source of renewable material on earth. This material can be found in agricultural residues, forestry waste, municipal solid waste, woods, and grasses, making them widely available at low cost, which is advantageous to the industrial context (Palmqvist and Hahn-Hägerdal, 2000b; Dahlman et al., 2003). Hemicellulose is a heterogeneous group of polysaccharides that comprises 15–35% of plant biomass (Gírio et al., 2010). Achieving 31.4% in switchgrass (Sun and Cheng, 2002), 29.3% in willow (Jorgensen et al., 2007), 28.6% in sugarcane bagasse (Fernandes Pereira et al., 2011), 22.1% in corn stover (Jorgensen et al., 2007), 19.7% in birchwood (Jorgensen et al., 2007) and 18% in spruce (Tengborg et al., 1998). Besides different amounts, the distribution of hemicellulose varies significantly between different plants. Depending on the source of the biomass (softwoods or hardwoods), its structure and composition can also vary. Softwood hemicellulose (pine and spruce, for example) presents a higher proportion of mannose and glucose units than hardwood hemicellulose (such as *Eucalyptus*, willow, and oak), which in turn, has a higher ratio of xylose units typically acetylated (Palmqvist and Hahn-Hägerdal, 2000b; Dahlman et al., 2003). The dominant hemicellulose polymer in hardwood biomass, xylan, is composed of repeating β (1–4)-linked xylose residue backbone, with acetyl and (methyl)glucuronic acid side groups. However, variations exist in its structures between different species (Rennie and Scheller, 2014; Wierzbicki et al., 2019).

Until recently, xylan represented the main component of plant biomass that cannot be efficiently utilized for biofuels production by fermentation using modified *S. cerevisiae* yeast strain. However, in 2004, Katahira and coauthors first demonstrated that a xylose-consuming *S. cerevisiae* strain expressing xylanolytic enzymes was able to produce ethanol from hemicellulose fraction although in lower levels (Katahira et al., 2004). Xylo-oligosaccharides (XOS)-consuming *S. cerevisiae* strains can represent an essential step to reach a more cost-effective second-generation ethanol production, conferring three significant advantages: 1) less intensive pre-treatment conditions would be required – harsh lignocellulosic pretreatment has been applied to release monomers (fermentable sugars), however during this process several yeast growth inhibitors are formed, such as furans, organic acids, phenols, and inorganic salts. Different aspects can interfere with the severity of the pretreatment process, which include holding time, pH, and temperature (Pedersen and Meyer, 2010). The lower severity process can result in high amounts of oligosaccharides, lower monosaccharides, and lower inhibitors compounds, as presented by Brenelli et al. (2020). In their study, the authors evaluated the effect of a mild deacetylation treatment accomplished by hydrothermal pretreatment of raw sugarcane straw and achieve 81.5% of soluble hemicellulose with XOS yields up to 9.8% (w/w of an initial straw). These investigators found that an increase in the pretreatment temperature from 180 to 210°C, achieving a severity factor greater than 4, was accompanied by an increase in xylose production and lower oligosaccharides production. Under a lower severity factor condition (3.95) the crude hydrolysate yielded approximately 13.5 g L^−1^ soluble XOS as well low amounts of arabinose, xylose, formic acid, acetic acid, and furfural were obtained. Increased temperature is related to an increase in the severity of the treatment, resulting also in the formation of inhibitors for both the enzymatic and fermentation processes (Pedersen and Meyer, 2010). It is worth mentioning that the depolymerization of cellulose and solubilization of hemicellulose and lignin vary according to the proposed pretreatment process and the severity factor applied in the respective process (Lynd et al., 2002). Moreover, the preparation of hemicellulose hydrolysate includes acid addition, high pressure and temperature which cause environmental pollution and equipment corrosion; therefore, successful ethanol production through XOS fermentation would make the process more environmentally friendly (Woodward and Wiseman, 1982; Gueguen et al., 1997; Nevoigt, 2008; Li et al., 2013); 2) lower demand for xylanolytic enzymes would be required, achieving lower production costs—the biomass enzymatic hydrolysis is a crucial step in the overall process due to its relatively large contribution to the total cost of lignocellulosic-derived ethanol (Nieves et al., 1997; Galbe and Zacchi, 2002); to maximize xylose yield and minimize the production of inhibitors, higher amounts of xylanolytic enzymes are required for total degradation of xylan and XOS which is prohibitively expensive on an industrial scale (Galbe and Zacchi, 2002), milder pretreatment methods have been described and 3) industrial competitive advantages (mainly for recombinant microorganism which are able to uptake and consume XOS internally)–it is expected that XOS-consuming *S. cerevisiae* strains would have a competitive advantage concerning other microorganisms, such as contaminating bacteria and wild *Saccharomyces* and non-*Saccharomyces* species that naturally use xylan as carbon source (Cabrini and Gallo, 1999; Amorim et al., 2011). It is important to point out that, in order to obtain a second-generation ethanol cost-competitive with first-generation ethanol, it is crucial to obtain microorganisms with unique genotype features to hydrolyze hemicellulose internally through recombinant DNA technology, which represents the best option to overcome the barriers to the commercial exploitation of lignocellulosic bioethanol.

Heterologous expression of xylose and XOS-producing enzymes in *S. cerevisiae* has been extensively reported. However, only one study has reported an *S. cerevisiae* strain able to break xylan down in an intracellular environment (Li et al., 2015). For this reason, although there are engineering efforts to improve direct xylan utilization by this microorganism, some limitations still remain, such as the affinity between XOS and cell membrane transporters, and the understanding of metabolic pathways regulation. This overview examines all strategies reported to date adopted for the re-construction of XOS assimilation in *S. cerevisiae* yeast strains, focusing on those that bioethanol could be bio-converted from hemicellulose fraction.

## Latest Trends in Xylose-Utilizing *S. cerevisiae*

Although *S. cerevisiae* strains present all genes required for the xylose fermentation, i.e., xylose reductase (*XR*), xylitol dehydrogenase (*XDH*), and xylulokinase (*XKS1*), only *XKS1* has been functionally expressed. *XKS1* phosphorylates xylulose into xylulose-5-phosphate which is introduced into the central metabolism through the pentose-phosphate pathway. Previous studies have reported that the wild type of *S. cerevisiae* is capable of naturally assimilating xylulose as a sole carbon source, although at a low rate, under aerobic conditions. However, xylulose is a rare pentose not widely available in nature and probably due to this, the challenge of directing xylulose fermentation by *S. cerevisiae* has received little attention (Jeffries, 1983; Eliasson et al., 2000; Mittelman and Barkai, 2017; Patiño et al., 2019). Furthermore, xylose fermentation by *S. cerevisiae* requires additional interventions in endogenous genes expression and/or kinetic properties (Patiño et al., 2019).

With a focus on second-generation bioproducts, it is not surprising that many studies have attempted to develop laboratory and industrial engineered *S. cerevisiae* strains capable of simultaneous glucose and xylose fermentation by the expression of heterologous xylose consumption genes (Eliasson et al., 2000; Kuyper et al., 2005; Kwak and Jin, 2017; Li et al., 2019). Many studies have shown that different mutations can improve xylose fermentation by yeast. In Table 1 we benchmark five xylose-utilizing strains with superior ethanol yields on xylose metabolism.

**TABLE 1:** Literature data on engineered, xylo-oxidoreductase and xylose-isomerase -based *S. cerevisiae* strains.

Strain	Parental strain	Relevant genotype/features	Culture conditions	Xylose specific consumption rate	Ethanol production rate	Ethanol productivity	Ethanol yield (g g_xylose_^−1^)	Reference
LVY34.4	PE-2 (*MATα*)	XI–*Orpinomyces* sp. *XYLA*, *XKS1*, *TAL1*, *RKI1*, *TKL1*, *RPE1*, *Δgre3*, evolved	Microaerobic batch, YPX, 3% xylose, ICW 0.25 g DCW L^−1^	1.320 (g g^−1^ h^−1^)	0.620 (g g^−1^ h^−1^)	ND	0.460	Dos Santos et al. (2016)
XUSE	BY4741 (*MATα his3 leu2 met15 ura3*)	XI–*Piromyces* sp. *XYLA*, *XKS1*, *TAL1*, *∆gre3*, *∆pho13*, evolved	Microaerobic batch, YSC, 2% xylose, OD_600_ 10	ND	ND	ND	0.400	Tran Nguyen Hoang et al. (2018)
IMU078	CEN.PK113-5D (*MATa ura3*)	XI—*Piromyces* sp. *XYLA*, *RPE1*, *RKI1*, *TAL1*, *TKL1*, *NQM1*, *TKL2*, *XKS1*, *∆gre3*	Anaerobic batch, Synthetic medium with L-aspartate instead of ammonium sulfate, 2% xylose, ICW 0.02 g DCW L^−1^	ND	ND	ND	0.406	Bracher et al. (2019)
SR8N	D425-2 (*MATa his3 leu2 ura3*)	XR/XDH–*S. stipitis XYL1*, *XYL2* and *XYL3*, *Lactococcus lactis NoxE*, *Δpho13*, *Δald6*	Microaerobic batch, YNB, 4% xylose, OD_600_ 10	ND	ND	1.220 (g L^−1^ h^−1^)	0.391	Kim et al. (2013b), Zhang et al. (2017b)
YRH1490	PE-2 (*MATα*)	XR/XDH–*S. stipitis XYL1* and *XYL2*, *XKS1*	Microaerobic batch, YPX, 8% xylose, OD_600_ 1	ND	ND	0.310 (g L^−1^ h^−1^)	0.330	Dias Lopes et al. (2017)

ICW, initial cell weight.

OD_600_ = Initial OD_600_.

ND, no data available.

**FIGURE 1:**
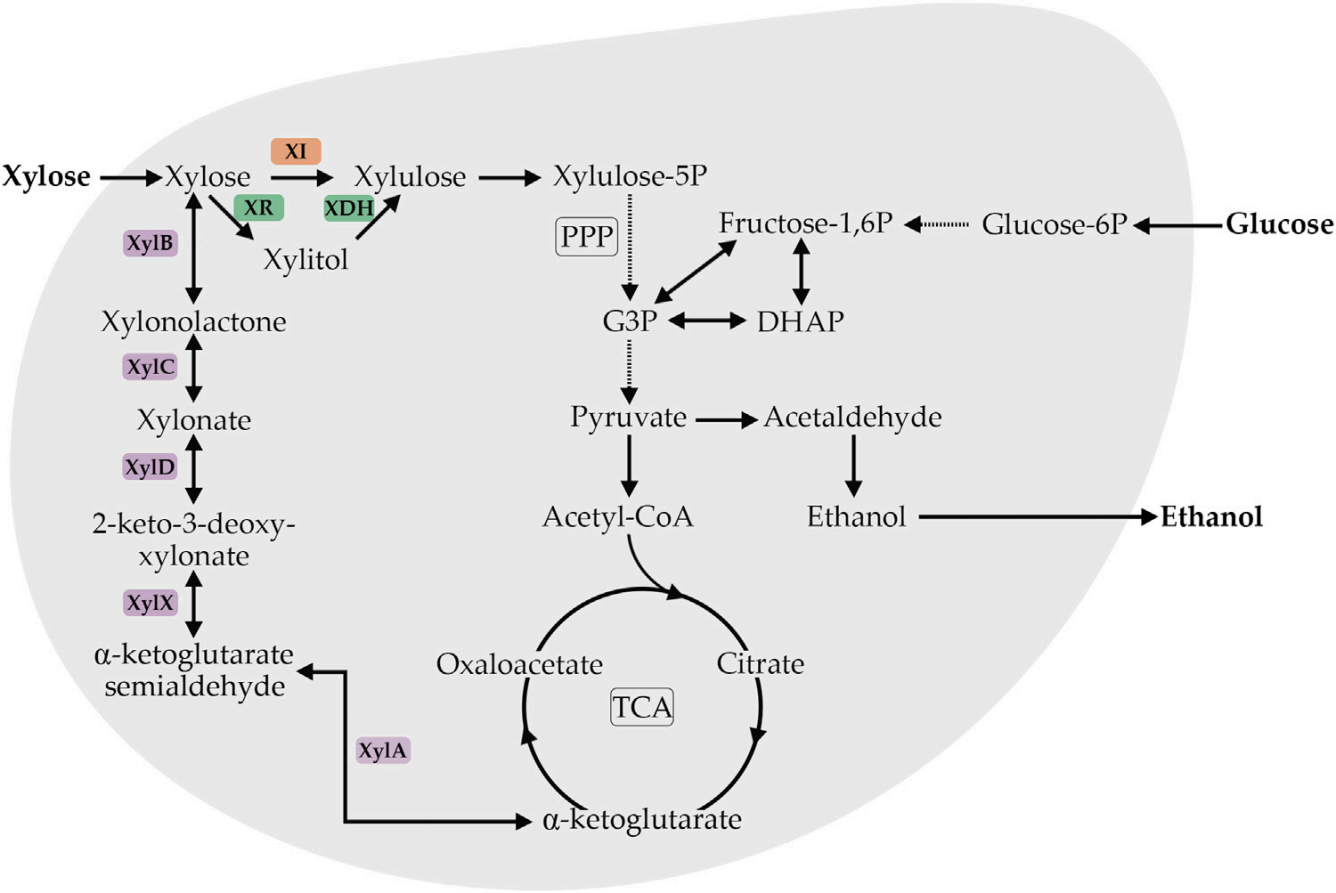
Schematic overview of the xylose degradation pathway associated with the central carbon metabolism in yeast. The orange box indicates the redox-cofactor-independent xylose isomerase (XI), red boxes indicate the pyridine-nucleotide-dependent xylose reductase (XR) and xylitol dehydrogenase (XDH). Purple boxes indicate the five reaction steps of the Weimberg pathway catalyzed by xylose dehydrogenase (XylB), xylonolactonase (XylC), xylonate dehydratase (XylD), 3-keto-2deoxy-xylonate dehydratase (XylX), and α-ketoglutarate semialdehyde dehydrogenase (XylA). Abbreviations: PPP—pentose phosphate pathway, TCA—tricarboxylic acid cycle, G3P—glyceraldehyde 3-phosphate, DHAP—dihydroxyacetone phosphate. Adapted from Borgström et al. (2019).

In nature, pentose assimilation is widespread across many prokaryotes and eukaryotes, such as *Pseudomonas fragi* (Weimberg, 1961), *Kluyveromyces lactis* (Margaritis and Bajpai, 1982), *Scheffersomyces stipitis* (Toivola et al., 1984), *Candida shehatea* (Toivola et al., 1984), *Pachysolen tannophilus* (Smiley and Bolen, 1982; Toivola et al., 1984), *Trichoderma* sp. (Kulkarni et al., 1999), *Aspergillus* sp. (Kulkarni et al., 1999), *Cryptococcus adeliae* (Petrescu et al., 2000), *Pseudoalteromonas haloplanktis* (Van Petegem et al., 2002), *Hansenula polymorpha* (Ryabova et al., 2003), *Bacillus halodurans* (Honda and Kitaoka, 2004), *Bacillus subtilis* (Collins et al., 2006), *Caulobacter crescentus* (Stephens et al., 2007), *Plectosphaerella cucumerina* (Zhang et al., 2007), *Haloferax volcanii* (Johnsen et al., 2009), *Aurebasidium pullulans* (Yegin, 2017). To date, three different pathways for xylose assimilation have been identified in these microorganisms and they are differentiated by the involvement of a phosphorylation step (Figure 1). In the first possibility, xylose is isomerized to xylulose and then phosphorylated to form xylulose-5-P. Two metabolic pathways have been identified which involve this strategy: the redox pathway, involving the combined activity of pyridine-nucleotide-dependent xylose reductase (XR) and xylitol dehydrogenase (XDH), and the isomerization pathway involving the redox-cofactor-independent xylose isomerase (XI). The main difference between these pathways is the dependence on cofactors (oxido-reduction pathway) or not (isomerization pathway). Both metabolic pathways have been used extensively as targets in engineered *S. cerevisiae* and have been reviewed in detail (Kim et al., 2012, Kim et al., 2013; Harner et al., 2015; Kwak and Jin, 2017; Bracher et al., 2019). The generation of xylulose-5-phosphate via the oxidoreductase pathway allows a link to glycolysis, the central carbon flux, through the non-oxidative part of the pentose phosphate pathway (Stincone et al., 2015). Optimized *S. cerevisiae* recombinant strains overexpressing XI or XR/XDH have been reported (Table 1). The success of these strategies enables new perspectives on the carbon-source range assimilated by *S. cerevisiae* to be considered. Since xylose assimilation by engineered *S. cerevisiae* strains has become well-established, new approaches have been adopted to enable *S. cerevisiae* to consume XOS instead of xylose and glucose (La Grange et al., 2000, 2001; Fujita et al., 2002; Qian et al., 2003; Katahira et al., 2004; Lee et al., 2009; Sun et al., 2012; Li et al., 2015; dos Reis et al., 2016; Sekar et al., 2016; Zhang et al., 2017a). Scientific interest in this field is increasing steadily, but still much must be done to obtain an efficient XOS-consuming *S. cerevisiae* strain.

An additional xylose assimilation possibility is the so called Weimberg pathway, which is characterized as an oxidative but non-phosphorylating metabolic pathway without xylose to xylulose isomerization. This pathway received much less attention when compared with oxido-reductase and isomerase pathways but recently has gained major attention from research groups (Shen et al., 2020). This pathway provides an alternative entry point for xylose into yeast central metabolism with possibilities to produce new compounds that are intermediates or derivatives from the TCA cycle and provides an interesting route for the production of xylose-derived α-ketoglutarate (Figure 1) (Weimberg, 1961). Recently, the Weimberg pathway enzymes derived from *Caulobacter crescentus* and *Corynebacterium glutamicum* were functionally expressed in *S. cerevisiae*; however, pathway intermediates were detected, indicating that this pathway needs further optimization (Borgström et al., 2019). Some of the drawn hypotheses could explain this incompatibility between prokaryotic and eukaryotic proteins, such as deficiency of enzymatic cofactors, posttranslational modifications of the protein, differences in the internal pH of the parental and the host cell (Sarthy et al., 1987), and the improper folding of the protein (Gárdonyi and Hahn-Hägerdal, 2003; Xia et al., 2016).

## Xylanolytic Enzyme Systems

The most selective method for the conversion of poly- to monosaccharides is by using enzymes. Most studies on hemicellulases have focused on xylanolytic enzymes, which are responsible for xylan hydrolysis (Gírio et al., 2010). The study of microorganisms able to hydrolyze xylan started more than 130 years ago, probably in 1889 by Hoope-Seyler (Whistler and Masak Jr., 1955). Since then, many organisms with the ability to colonize and grow on plant biomass have been identified. Xylanolytic enzyme producers are widespread, such as fungi, bacteria, yeast, marine algae, protozoans, snails, crustaceans, and insects, of which bacterial and fungal xylanases have the most important role concerning heterologous expression in *S. cerevisiae* (Jeffries, 1983; Biely, 1985; Kulkarni et al., 1999; Katahira et al., 2004; Schmoll and Schuster, 2010). *Trichoderma reesei*, *Trichoderma atroviride*, *Trichoderma virens* (Beg et al., 2001), *Aspergillus niger* (La Grange et al., 2001), *Neurospora crassa* (Li et al., 2015), *Aspergillus foetidus* (Whistler and Masak Jr., 1955), *Bacillus pumilus* (Pan et al., 1991) are some examples of potent xylanolytic enzymes producers. These enzymes have potential for the application of xylanases in several industries, such as in the pulp and paper, food additives, animal feed, textiles, drinks industries, ethanol, and xylitol production (Polizeli et al., 2005). The search for newer microbial xylanases producers is ongoing, together with molecular biology studies on the regulation of xylanases expression and their heterologous expression in non-xylanolytic microorganisms.

**FIGURE 2:**
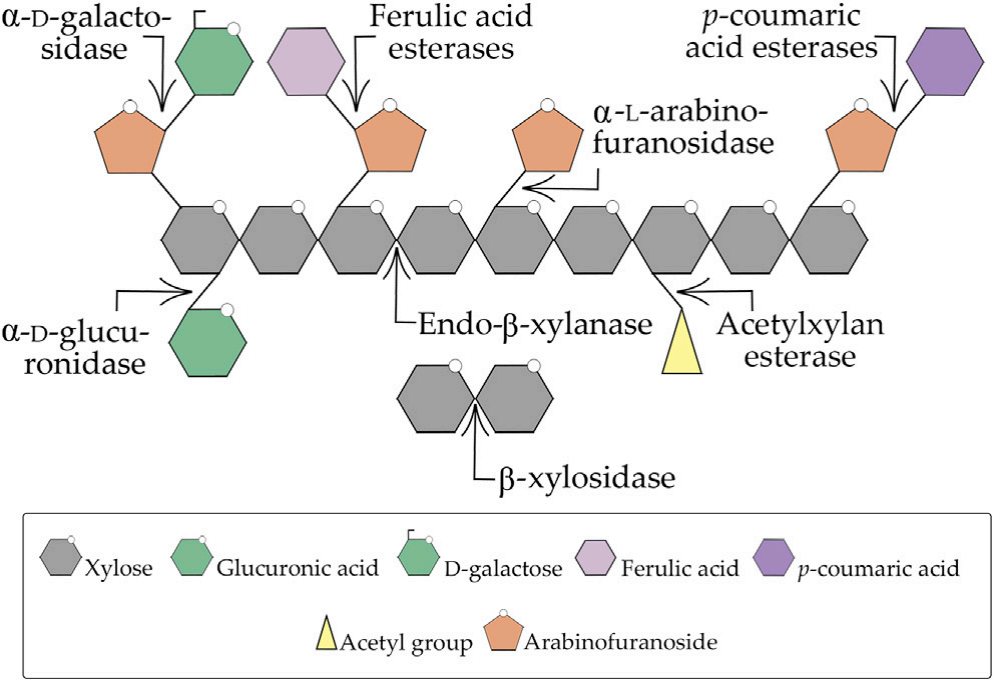
Xylan degradation scheme. The arrows represent each enzyme activity for a determined substrate. Adapted from Bhardwaj et al. (2019).

Xylans represent a family of complex non-cellulosic branched polysaccharides that consists structurally of linear homopolymeric β-(1,4)-xylopyranosyl units with a diversity of substituted groups, which vary quantitatively and qualitatively according to the plant or the method of isolation. They can be comprised of 4-*O*-methyl-D-glucuronopyranosyl, α-L-arabinofuranosyl, acetyl, feruloyl and/or *p*-coumaroyl groups (Figure 2) (Wong et al., 1988; Collins et al., 2005; Biely et al., 2016). Wood xylan exists as *O*-acetyl-4-*O*-methylglucuronoxylan in hardwoods and as arabino-4-*O*-methylglucuronoxylan in softwoods, which represent the two major forms of xylan in wood, whereas xylans in grasses and annual plants are typically arabinoxylans (Kulkarni et al., 1999). On the other hand, in esparto grass, tobacco stalks, and guar seed husk another type of xylan has been identified, the homoxylans which are composed exclusively of xylosyl residues (Sunna and Antranikian, 1997).

Due to the heterogeneity and complex chemical nature of xylan, its complete breakdown requires the action of a consortium of enzymes with diverse specificities and modes of action (Figure 2). Thus, it is not surprising that xylan-degrading organisms produce a multienzyme system of xylanases that present diverse structures, different mechanisms of action, substrate specificities, hydrolytic activities, and physicochemical characteristics (Beg et al., 2001; Collins et al., 2005; Moreira and Filho, 2016). It is interesting to note that microorganisms can produce multiple forms of the same xylanase family, showing that some factors such as differential processing of mRNA, post-translational modification, proteolytic digestion, and differential expression by distinct alleles of one gene, or even by completely separate genes affect this multiplicity of xylanases secreted (Polizeli et al., 2005; de Vries et al., 2017).

The carbohydrate-active enzymes (CAZy—www.cazy.org) database collectively complies and assigns xylanases are glycoside hydrolases (GH) that catalyze the hydrolysis of 1,4-β-D-xylosidic linkages in xylan. Sequence-based classification has grouped xylanases in two major families GH10 and GH11, but xylanases are also found in other GH families, 3, 5, 8, 9, 10, 11, 12, 16, 26, 30, 43, 44, 51, 62, 98, and 141 (Collins et al., 2005; Chakdar et al., 2016; Velasco et al., 2019). GH10 members are highly active on short XOS, thereby indicating small substrate-binding sites. The major enzymes of this family are endo-β-1,4-xylanases (Collins et al., 2005). In contrast, the enzymes that belong to the GH11 family are most active on long-chain XOS. Furthermore, this family is monospecific, which means they are exclusively active on D-xylose-containing substrates (Collins et al., 2005; Chakdar et al., 2016). Some genera of fungi and bacteria produce more than one subfamily of xylanases. However, the majority of the bacterial xylanases belong to the GH10 family, whereas fungal xylanases majorly belong to the GH11 family (Liu et al., 2011).

There is a great diversity of xylanases produced among bacterial genera with *Bacillus* presenting a potential source of these enzymes (Subramaniyan and Prema, 2002; Gupta and Verma, 2015; Chakdar et al., 2016). Xylanases obtained from bacterial sources are known to be active and stable in a wide range of pH and temperature, such as temperature from 30 to 60°C, pH from 5.0 to 9.0 (See some examples in Table 2). These enzymes are produced alone mostly, thereby reducing the downstream process time (Chakdar et al., 2016).

**TABLE 2:** Characteristics of xylanase from different microorganisms functionally expressed in *S. cerevisiae*.

Organism	Enzyme	Cloning process	Host		Optimum		Activity in *S. cerevisiae*	Reference
			Name	Remarks	Temperature	pH		
Ethanol production has not been reported								
**Bacterial species**								
*B. pumilus* PLS	β-xylosidase	pDLG12—2µ yeast plasmid	*S. cerevisiae* Y294	*MATα leu2-3 112 ura3-52 his3 trip1-289*	45–50°C	6.6	0.09 nkat ml^−1^	La Grange et al. (1996, 1997)
*B. pumilus* IPO	Xylanase (*xynA*)	pNAX2—2µ yeast plasmid	*S. cerevisiae* NA87-11A cir^+^	*ho MATα leu2-112 pho3 pho5 his3 trip1*	40°C	6.5	0.36 U (mg protein)^−1^	Panbangred et al. (1983), Pan et al. (1991)
*B. pumilus* IPO	β-xylosidase (*xynB*)	pYXB—2µ yeast plasmid	*S. cerevisiae* NA87-11A cir^+^	*ho MATα leu2-112 pho3 pho5 his3 trip1*	ND	ND	0.28 U (mg protein)^−1^	Pan et al. (1991)
*Caldocellum saccharolyticum*	Xylanase (*xynA*)	pFGxyn—2µ yeast plasmid	*S. cerevisiae* STX329-3A	*MATα ade1 his2 trip1 gal2*	ND	ND	90 U (mg protein)^−1^	Donald et al. (1994)
*Bacillus* sp. KK-1	β-xylosidase (*xylB*)	pBX45—2µ yeast plasmid	*S. cerevisiae* SEY2102	*MATα ura3-52 leu2-112 his4-519 suc2-Δ9*	ND	ND	2.9 U mL^−1^	Kim et al. (2000)
*B. pumilus* PLS	Xylanase (*xynA*)	pFN3—2µ yeast plasmid	*S. cerevisiae* Y294	*MATα leu2-3 112 ura3-52 his3 trip1-289 Δfur*	58°C	6.2	8.5 nkta mL^−1^	Nuyens et al. (2001)
		pFN4 - 2µ yeast plasmid			58°C	6.2	4.5 nkta mL^−1^	
*Penicillium purpurogenum* ATCC No. MYA-38	Xylanase (*xynA*)	pYEplac181—integrating plasmid	*S. cerevisiae* YM335::RY171	*Mata gal4-536 ura3-52 ade2-101 lys2-801 his3-200 met Δgal*	ND	ND	4.52 U mL^−1^	Ma and Ptashne (1987), Chávez et al. (2002)
*Bacillus* spp*.*	Xylanase (*xynB*)	pAGX3—2µ yeast plasmid	*S. cerevisiae* SEY2102	*MATα leu2-112 ura3-52 his4-519 suc2-Δ9*	ND	ND	7.56 U mL^−1^	Lee et al. (2007)
*Bacillus* spp*.*	Xylanase (*xynB*)	pADEX-1—2µ yeast plasmid	*S. cerevisiae* SEY2102	*MATα ura3-52 leu2-112 leu2-3 his4-519 suc2-Δ9*	ND	ND	9.8 U mL^−1^	Lee et al. (2009)
*Bacillus* sp. HY-20	Xylanase (XylP)	pGMF-xylP—2µ yeast plasmid	*S. cerevisiae* SEY2102	MATα leu2-3 112 ura3-52 his4-519 suc2-Δ9	ND	ND	70.1 U mL^−1^	Kim et al. (2013a)
*Bacillus* sp. HY-20	Xylanase (XylP)	pGMF-xylP—2µ yeast plasmid	*S. cerevisiae* FY833	MATa leu- Δ 1 ura3-52 his3- Δ200 lys2-Δ202 trp1- Δ63	ND	ND	42.4 U mL^−1^	Kim et al. (2013a)
*Bacillus* sp. HBP8	Xylanase (XynHB)	pHBM367H—rDNA-mediated integration plasmid	*S. cerevisiea* INV	MATa his3D1 leu2 trp1-289 ura3-52 his3D1 leu2 trp1-289 ura3-52	ND	ND	255 U (g DWcell)^−1^	Fang et al. (2017)
**Fungal species**								
*Cryptococcus albidus*	Xylanase (XLN)	pVT100—2µ yeast plasmid	*S. cerevisiae* T109-3C	MATa Cir+ leu2-3 leu2-112 his3-11 his3-5 ra3 can1	ND	ND	1.3 U (mg protein)^−1^	Moreau et al. (1992)
*Aspergillus kawachii* IFO4308	β-xylosidase (xynC)	pJC1—2µ yeast plasmid	*S. cerevisiae* Y294	MATα leu2-3 112 ura3-52 his3 trip1-289	60°C	3	300 nkat ml^−1^	Crous et al. (1995)
*Aureobasidium pullulans* Y-2311–1	Xylanase II (XynA)	2µ yeast plasmid	*S. cerevisiae* INVSc1	MATα his3-Δ1 leu2 trp1-289 ura3-52	ND	ND	32.9 U ml^−1^	Li and Ljungdahl, (1996)
*A. nidulans* G191	Xylanase (xlnA)	pYLA1—2µ yeast plasmid	*S. cerevisiae* OL1	MATa leu2-3 112 his3-11 15 ura3-251 337	ND	ND	65 U ml^−1^	Pérez-González et al. (1996)
	Xylanase (xlnB)	pYLB1—2µ yeast plasmid			ND	ND	25 U ml^−1^	
*T. reesei* RutC-30	α-Arabinofuranosidase (abfB)	p17SA—2µ yeast plasmid	*S. cerevisiae* DBY746	MATα his3 Δ1 leu2-3 112 ura3-52 trp1-289 cyhR	ND	ND	171.1 nkat ml-1	Margolles-Clark et al. (1996)
*A. niger* ATCC 90196	β-xylosidase	pMLU1—2µ yeast plasmid	*S. cerevisiae* Y294	MATα leu2-3 112 ura3-52 his3 trip1-289 Δfur1	60°C	4	91 nkat ml^−1^	Luttig et al. (1997)
*T. reesei*	Xylanase II (XYNII)	pCAS1—2µ yeast plasmid	*S. cerevisiae* MT8-1	MATα ade leu2 ura3 his3 trip1	40°C	5	1.78 µmol min^−1^ (g DWcell)^−1^	Fujita et al. (2002)
*Trichoderma* spp.	Endoglucanase (GenBank access No. AY466436)	pAGX1—2µ yeast plasmid	*S. cerevisiae* SEY2120	MATα leu2-112 ura3-52 his4-519 suc2-Δ9	ND	ND	0.6 U ml^−1^	Lee et al. (2007)
*C. flavus* I-11	Xylanase (*CfXYN1*)	Yep351PGK—2µ yeast plasmid	*S. cerevisiae* MFL	leu2	50°C	3	2.5 U ml^−1^	Parachin et al. (2009)
A. niger IME-216	Xylanase	pUPXR—integrating plasmid	*S. cerevisiae* YS2_2	Industrial ethanol producing strain	ND	ND	74.8 U ml^−1^	Tian et al. (2013)
Ethanol production has been reported								
**Fungal species**								
*A. orizae* NiaD300	β-xylosidase (XylA)	pUCSXIIXA—cell-surface expressing plasmid	*S. cerevisiae* MT8-1	MATa ade leu2 his3 ura3 trp1 SsXYL1 SsXYL2 ScXKS1	ND	ND	234 U (g DWcell)^−1^	Katahira et al. (2004)
*T. reesei* QM9414	Xylanase II (XYNII)				ND	ND	16 U (g DWcell)^−1^	
*T. reesei* QM9414	β-xylosidase	pAUR-XSD—2µ yeast plasmid	*S. cerevisiae* MA-D4	MATα leu2 his3 ura3 can1 SsXYL1 SsXYL2 ScXKS1 Δaur	ND	ND	6 nmol min^−1^ (mg protein)^−1^	Fujii et al. (2011)
*T. reesei*	β-xylosidase (XYL)	pUCSXylAf—integrating plasmid	*S. cerevisiae* OC-2	MATa/α SsXYL1 SsXYL2 ScXKS1	60°C	ND	ND	Saitoh et al. (2011)
*T. reesei*	β-xylosidase (*XYNII*)	pδW-GPAGXynII- integrating plasmid	*S. cerevisiae* MT8-1	Mata ade his leu2 trip1 ura3 SsXYL1 SsXYL2 ScXKS1	ND	ND	41.2 U (g DWcell)^−1^	Sakamoto et al. (2012)
*A. oryzae*	β-xylosidase (*XylA*)	pIHBGXylA—integrating plasmid			ND	ND	16.8 U (g DWcell)^−1^	
*T. reesei* DSM769	Xylanase II (XynII)	pYD1—2µ yeast plasmid	*S. cerevisiae* EBY100 (Invitrogen, Carlsbad, CA)	SsXYL1 SsXYL2 SsXYL3	ND	ND	ND	Sun et al. (2012)
*A. niger* DSM821	β-xylosidase (*XlnD*)				ND	ND	ND	
*A. niger* DSM821	α-arabinofuranosidase (*AbfB*)				ND	ND	ND	
A. terreus	Xylanase	pRSK2—2µ yeast plasmid	*S. cerevisiae* INVSc1	MATa his3Δ1 leu2 trp1-289 ura3-52 CtXR	ND	ND	ND	Li et al. (2013) ^a^
	β-xylosidase							
*N. crassa* FGSC 2489	β-xylosidase (GH43-2)	pXD8.7—2µ yeast plasmid	*S. cerevisiae* SR8U	MATα ura3 SsXYL1 SsXYL2 SsXYL3 Δpho13 Δald6	ND	7	ND	Kim et al. (2013b), Li et al. (2015)
	β-xylosidase (GH43-7)							
*U. bevomyces*	Xylanase 1 (XNA1)	P423—2µ yeast plasmid	*S. cerevisiae* YSX3 Δhis	Mata trp1 can1 cyn1 gal+ leu2::LEU2-TDH3P-PsXYL1-TDH3T ura3::URA3-TDHP-PsXYL2-TDH3T Ty3::G418-PsXYL3 YOR202w::hphNT1	ND	ND	ND	Lee et al. (2015)
	Xylosidase 2 (XD2)	P424—2µ yeast plasmid			ND	ND	ND	
	Arabinofuranosidase (ABF)	P424—2µ yeast plasmid			ND	ND	ND	
*T. reesei* QM6a	Endoxylanase (Xyn2)	pVSDis-TrXyn2—cell-surface expressing plasmid	*S. cerevisiae* EBY100	Mata AGA1::GAL1-AGA1::URA3 ura3-52 trp1 leu2-Δ200 his3-Δ200 pep4::HIS3 prb11.6R can1 GAL1 PrXI PrXKS	ND	ND	1.197 U mg^−1^	Tabañag et al. (2018)
	β-xylosidase (Bxl1)	pVSDis-TrBxl1—cell-surface expressing plasmid			ND	ND		
	Acetyl esterase (Axe1)	pVSDis-TrAxe1—cell-surface expressing plasmid			ND	ND		
	α -glucuronidase (Glr1)	pVSDis-TrGlr1—cell-surface expressing plasmid			ND	ND		
	α-arabinofuranosidase (Abf1)	pVSDis-TrAbf1—cell-surface expressing plasmid			ND	ND		

a The final goal of this work was xylitol production from xylan. Li et al. (2013) achieved a xylitol yield of 0.71 g xylitol (g xylan)^−1^, and *S. cerevisiae* recombinant strain, Sc-K2, produced 1.94 g L^−1^ xylitol when cultivated in YPD supplemented with 3 g L^−1^ xylan.

Ss, *S. stipitis*; Sc, *S. cerevisiae*; Ct, *Candida tropicalis*; Pr, *Prevotella ruminicola*.

ND, no data available.

Despite the great diversity of bacterial xylanase producers, filamentous fungi are the major commercial source due to their higher levels of xylanase secretion (Kulkarni et al., 1999; Polizeli et al., 2005). Some works have demonstrated that many fungal species produce xylanase when cultured on cellulose (Mishra et al., 1984; Biely, 1985; Wong et al., 1988), perhaps because the cellulose substrates contain traces of hemicellulose (Polizeli et al., 2005). Nevertheless, in an opposite scenario, several fungal species produce specific xylanases with little or no cellulase background in the presence of xylan, which indicates the xylanolytic and cellulolytic systems are likely to be under separate regulatory control (Biely, 1985; Wong et al., 1988). And interestingly XOS reduced the efficiency of cellulose hydrolysis by cellulase (Zhang et al., 2012; Wang et al., 2018) which cannot be effectively relieved by increasing the loading of the cellulose substrate or cellulase (Wang et al., 2018). However, some fungi species require low pH for the growth and production of xylanases which necessitates additional steps in the subsequent stages which make fungal xylanases less attractive (Chakdar et al., 2016).

Among xylanases, endo-β-xylanases (xylanase or endo-β-1,4-xylanases) and β-d-xylosidases (β-xylosidases or xylosidase) have been most extensively studied. Endo-β-xylanases (EC 3.2.1.8) randomly cleave the β-1,4 linkages between the xylopyranosyl units from the xylan backbone, producing mixtures of XOS (Biely, 1985; Kulkarni et al., 1999). β-d-xylosidases (EC 3.2.1.37) are known to be the major component of xylanase systems. They are produced by biodegradative microorganisms to hydrolyze XOS releasing d-xylose; however, usually, they do not hydrolyze xylan, with their best substrate being xylobiose and their affinity for XOS being inversely proportional to its degree of polymerization. They act on the non-reducing ends of their substrate, XOS and/or xylobiose (Wong et al., 1988; Collins et al., 2005; Polizeli et al., 2005). A high concentration of xylose in the fermentation broth can inhibit the activity of β-d-xylosidases (Fujii et al., 2011) which leads to the inefficient hydrolysis of hemicellulose and the accumulation of XOS and xylobiose by using microorganisms that do not consume xylose rapidly. Some β-d-xylosidases have been reported to possess α-L-arabinofuranosidase activity, e.g., the enzymes from *A. niger*, *T. reesei*, *T. ethanolicus*, and *Penicillium wortmannin* (Sunna and Antranikian, 1997). Other enzymes, such as α-L-arabinofuranosidase (EC 3.2.1.55), α-D-glucuronidases (EC 3.2.1.139), acetylxylan esterases (EC 3.1.1.72), ferulic acid esterases (EC 3.1.1.73), and *p*-coumaric acid esterases (EC 3.1.1.-) catalyze the removal of xylan side groups (Collins et al., 2005). All these enzymes act cooperatively to convert xylans into xylose, XOS, *O*-acetyl, L-arabinose, acetic and glucuronic acids (Beg et al., 2001; Polizeli et al., 2005).

Understanding the enzymes xylanolytic microorganisms produce for hemicellulose breakdown may become an important tool for re-construction of their XOS degradation pathway in non-xylanolytic microorganisms. As mentioned before, complete degradation of xylan is achieved by a variety of modular enzymes. Although many hemicellulolytic enzymes have been studied extensively, little is known about how microorganism cells sense the presence of xylan and uptake hemicellulose-derived products (Polizeli et al., 2005; Delmas et al., 2012; Najjarzadeh et al., 2020). The induction signal for the synthesis of xylanolytic enzymes is supposed to involve transporters of xylose and short XOS released by the action of little amounts of the enzymes produced constitutively, along with lactose, glucose, and even cellulose, that are able to cross the membrane and induce the regulatory machinery (Biely and Petráková, 1984; Royer and Nakas, 1989; Chandra Raj and Chandra, 1995; Christakopoulos et al., 1996a, 1996b; Kulkarni et al., 1999), which suggests a complex induction mechanism of xylanases. In their study, Delmas et al. (Delmas et al., 2012) studied the strategy of the filamentous fungus *A. niger* employ to degrade complex polysaccharides. They showed that wheat straw itself is not initially detected by the *A. niger*. According to their findings, the overall strategy appears to be an induction of a specific, small scale, sensory response by the onset of carbon starvation, mediated at least partially by alleviation of CreA-dependent catabolite repression, that triggers the release of a small subset of degradative enzymes which initiate degradation on a small scale, in turn releasing sugars that cause the fungus to express its full degradative arsenal (Delmas et al., 2012). More recently, Najjarzadeh and collaborators reported that xylotetraose is more effective than other substrates inducing endoxylanase, while xylohexaose and xylobiose are the best inducers of extracellular β-xylosidase, and cell-bound β-xylosidase, respectively (Najjarzadeh et al., 2020).

The driving force for xylose and xylo-oligomers uptake vary considerably among plant cell wall-degrading microorganisms. Several microbial transport systems show to be regulated by two-component systems, responding to environmental or intracellular signals to alter gene expression (Shulami et al., 2007). The two-component system includes two proteins, a receptor histidine kinase, and a response regulator. Each system uses transient phosphorylation of sensory system and a regulatory response of proteins at a specific histidine or aspartate residue for signal, and thus forms a pathway for phosphoryl transfer (Verhamme et al., 2002). Another example of oligomer transport system was described in bacterial species. Some members of the genus *Bifidobacterium* were found to be able to utilize xylan as a carbon source. A genome sequence analysis of these members have found a variety of genes related to ATP-binding cassette (ABC) sugar transporters (Liu et al., 2014; Chen et al., 2019a; Saito et al., 2020).

## Heterologous Expression of Xylanolytic Enzymes in the Yeast *S. cerevisiae*

Several reports have described the expression of heterologous endo-β-xylanases and β-xylosidase from both prokaryotes and eukaryotes in *S. cerevisiae* to enable the conversion of xylan or XOS into xylose by this species (Table 2). Usually, the complete hydrolysis of xylan requires at least these two enzymes (Biely, 1985): an endo-β-xylanase that cleaves xylan into XOS with diverse degrees of polymerization, followed by the breakdown of XOS to xylose by β-xylosidase (Wang et al., 2018). Although endo-β-xylanases are important for the hydrolysis process, β-xylosidase is considered a key enzyme (Banerjee et al., 2010), since XOS accumulation can reduce the efficiency of cellulases, such as cellobiohydrolase I (CBHI, from *Thermoascus aurantiacus*), cellobiohydrolase II (CBHII, from *Trichoderma reesei*) and endoglucanase II (from *T. aurantiacus*) (Zhang et al., 2012), which would affect conversion yields in simultaneous saccharification and fermentation (SSF) or consolidated bioprocessing (CBP). Therefore, reducing the concentration of XOS using β-xylosidase represents the best strategy to prevent enzyme inhibition.

The heterogeneous nature of hemicellulose represents a challenge for hemicellulase enzymes. Considering the first-generation ethanol industry, a huge amount of lignocellulose-based materials is formed during ethanol production, especially corn stover, sugarcane straw, and sugarcane bagasse, which are particularly attractive as second-generation ethanol feedstock. As mentioned earlier, their hemicellulose content can achieve up to 28% in sugarcane bagasse (Fernandes Pereira et al., 2011), 27–31% in sugarcane straw (Almeida and Colombo, 2021), and 22% in corn stover (Jorgensen et al., 2007). According to the chemical structure of hemicellulose, they present a very similar composition, which includes a high content of acetyl as side groups. Therefore, it is important to highlight that other enzymes would allow more efficient degradation of these hemicellulosic derived materials, such as acetylxylan esterases (EC 3.1.1.72) in combination with xylanase and xylosidase for hydrolyzing pretreated hardwood hemicellulose. The presence of acetyl group degrading enzymes may increase the accessibility of the xylose chain to xylanases.

## *S. cerevisiae* as a Platform to Produce Xylanolytic Enzymes

To the best of our knowledge, the first report of xylan-degrading genes expression in *S. cerevisiae* was in 1991, when Pan et al. (1991) described the expression of two enzymes, xylanase (a *xynA* gene product) and β-xylosidase (a *xynB* gene product) from *Bacillus pumilus* in yeast cells. Thereafter, physiological data related to the expression of the intracellular β-xylosidase from *B. pumilus* in *S. cerevisiae* has been published (Crous et al., 1995; Crous et al., 1996; La Grange et al., 1996; La Grange et al., 2000). A successful expression of a bacterial β-xylosidase from *B. pumilus* (*xynB*) in yeast cells was achieved by its fusion to a native secretion signal sequence named mating pheromone α-factor (MFα1s) (La Grange et al., 1997). The native open-reading frame of these enzymes starts with the codon TTG which is not recognized by *S. cerevisiae* for the initiation of translation. Even after replacing the TTG codon with an ATG starting codon, no β-xylosidase activity could be detected by the recombinant *S. cerevisiae* Y294 (La Grange et al., 1997).

Although there has been some success in functional expression of bacterial enzymes in *S. cerevisiae* (Table 2), this can be problematic, possibly because of incompatibility with eukaryotic chaperones (Sarthy et al., 1987; Gárdonyi and Hahn-Hägerdal, 2003; Xia et al., 2016). The heterologous expression of eukaryotic xylanases in *S. cerevisiae* naturally shows more compatibility since fungal species share many features, particularly related to transcription, translation, and protein stability (Frommer and Ninnemann, 1995). The first recombinant *S. cerevisiae* strain (namely IAF130) expressing heterologous eukaryotic xylanase was described by Moreau et al. (1992). However, because the cells could not catabolize xylose, the majority of the early reports of recombinant *S. cerevisiae* expressing β-xylosidases only demonstrated the conversion of xylan and XOS into xylose, xylobiose, and xylotriose, but not to ethanol.

Heterologous expression of the *T. reesei* xylanase II (*XYNII*) anchored on the cellular surface was described in the *S. cerevisiae* strain MT8 by using a cell surface engineering system based on α-agglutinin which consist of the fusion of the protein with the C-terminal-half region of an agglutinin. The recombinant strain MT8-1/pCAS1-XYNII was able to hydrolyze birchwood xylan into xylobiose and xylotriose. The proposed work did not aim to present a hydrolytic profile during the growth of MT8-1/pCAS1-XYNII in a medium containing complex sugar. Instead, xylanase activity was measured in both supernatant and pellet fractions from pre-cultured strain. XYNII activity was detected in the cell pellet with no leakage into the supernatant medium (Fujita et al., 2002).

## Ethanol Production From XOS by Extracellular Expression of Xylanolytic Enzymes in *S. cerevisiae* Strains

The first example of the concept of CBP applied to ethanol production from xylan using recombinant *S. cerevisiae* strain without the addition of exogenous xylan-degrading enzymes was described in 2004 (Katahira et al., 2004). In their work, xylanase II (*XYNII*) from *T. reesei* QM9414 and β-xylosidase (*XylA*) from *Aspergillus oryzae* NiaD300, were co-displayed on the cell surface of xylose-consuming *S. cerevisiae* harboring genes encoding the oxidoreductase pathway from *S. stipitis* and native xylulokinase (XKS) from *S. cerevisiae*. To obtain this strain, the C-terminal region of α-agglutinin was fused to both xylanolytic enzymes. The constitutive expression of *XYNII* and *Xyla* enabled xylan consumption and ethanol production without a lag-phase. The recombinant strain MT8-1/pUCSXIIXA/pWX1X2XK produced 7.1 g L^−1^ of ethanol after 62 h of fermentation in semi defined medium supplemented with birchwood xylan corresponding to 100 g of total sugar per liter as the sole carbon source. Despite the significant ethanol production, a large amount of xylan remained in the growth medium, suggesting that this strain needs further optimization.

Microbial surface display technology allows the expression of peptides and proteins on the surface of living cells in which proteins are expressed extracellularly, however, the enzymes remain fused at the cell with no leakage into the culture medium (Fujita et al., 2002; Katahira et al., 2004; Tafakori et al., 2012). Other examples of coexpression of xylanolytic enzymes anchored on the *S. cerevisiae* cellular surface considered the expression of bifunctional minihemicellulosomes, with several assembled modules included (Sun et al., 2012). Sun and coauthors constructed a recombinant yeast that directly produced ethanol from birchwood xylan through the expression of bifunctional minihemicellulosomes. This recombinant strain co-displays two complementary xylanases, *XYNII* and an *A. niger* xylosidase (*XDNL*) as well as a mini scaffolding (CipA3), which served as the basis to establish interaction between the enzymes and cell surface. According to their findings, the HZ3345 strain was able to ferment xylan into ethanol. The recombinant *S. cerevisiae* strain also contained an integrated xylose-utilizing pathway (XR, XDH, and XK from *S. stipitis*) to ensure the xylose assimilation. Interestingly, xylose production was immediately observed from XOS, without any lag phase, as previously observed (Katahira et al., 2004). The recombinant HZ3345 strain, produced 0.95 g L^−1^ of ethanol from approximately 3.0 g L^−1^ birchwood xylan after 80 h of cultivation under anaerobic conditions in YPBX (YP supplemented with birchwood xylan) supplemented with Tween and ergosterol.

In a more recent study, a blended bioprospecting approach was applied (Lee et al., 2015) along with rational and evolutionary engineering to improve xylan assimilation in an engineered xylan-catabolizing *S. cerevisiae* strain. The extracellular expression of xylan active enzymes (xylanase 1—XNA1, xylosidase 2—XD2, and arabinofuranosidase—AFB) from *Ustilago bevomyces* were cloned into 2-µm plasmids, p423 and p424 under the control of the *GPD* promoter. These plasmids were transformed into the *S. cerevisiae* YSX3 *Δhis3* strain, which has the xylose consumption pathway genes from *S. stipitis* integrated. Before applying the evolutionary approach, the recombinant strains grew slowly on xylan as a sole carbon source, producing 0.26 ± 0.008 g L^−1^ ethanol from YPXN (YP supplemented with 20 g L^−1^ xylan) after 5 days of cultivation. To improve its ability to assimilate xylan, serial-subcultures in the xylan medium were used over 3 weeks. After selecting clones with improved traits, the evolved strain was able to produce 23% more ethanol in complex media (YPXN, 2% xylan), 0.32 ± 0.028 g L^−1^. These results demonstrate the capacity to use whole-cell adaptive evolution to improve xylan metabolism by the cell.

The often-emphasized advantage of the xylose isomerase pathway in comparison with the oxidoreductase pathway was considered by Mert et al. (2016). In the earlier studies (Katahira et al., 2004; Fujii et al., 2011; Sun et al., 2012), engineered *S. cerevisiae* strains with XR/XDH were modified by the introduction of xylanolytic enzymes. Although the authors observed ethanol production from XOS, large amounts of xylose remained in the fermentation broth, probably resulting from a redox imbalance and/or inefficient xylose uptake; this, in turn, can inhibit β-xylosidase activity (Fujii et al., 2011; Peng et al., 2017; Niu et al., 2019). However, early attempts to express xylanases in engineered *S. cerevisiae* strains harboring XI had failed to produce ethanol from beechwood xylan (5%) as the sole carbohydrate source under aerobic growth over 28 days (Mert et al., 2016). The recombinant strain, Y294 [YMXI], which carries *T. reesei* endoxylanase (*XYNII*), *A. niger* β-xylosidase (*xlnD*), *S. stipitis* xylulokinase (*xyl3*), and the codon-optimized xylose isomerase encoding gene (*xylA*) from *Bacteroides thetaiotaomicron* was able to break down xylan into trisaccharide, disaccharides, and monosaccharides. However, the growth rate was low probably due to the low consumption of xylose. The small amounts of xylose consumed supported cell biomass synthesis only; ethanol, xylitol, glycerol, and acetic acid production were negligible. It is worth noting that when this recombinant strain Y294 [YMXI] was cultivated under similar conditions but using xylose (2%) as the sole carbon source, higher biomass was obtained, and larger amounts of xylose were consumed in a lower cultivation time. Moreover, xylitol production was also observed (Mert et al., 2016). Unfortunately, whether the expressed xylanolytic enzymes were secreted or expressed intracellularly in the Y294 [YMXI] strain is not clear. It is important to mention that in previous studies published by this research group, using a *S. cerevisiae* expressing an endoxylanase encoding gene (*xyn2*) and a xylosidase encoding gene (*xlnD*), enzyme activities were detected in the culture supernatant (La Grange et al., 2001), suggesting that these enzymes were secreted by the strain.

Recombinant gene expression can promote a nonspecific metabolic burden which reduces the maximum specific growth rate and production yield of the host, as previously observed (Görgens et al., 2001). In this research, *T. reesei* xylanase II (*XYN2*) was expressed in two recombinant *S. cerevisiae* strains, Y294 [PGK1-XYN] and Y294 [ADH2-XYN], using two 2-µm yeast plasmids under the control of either the yeast glycolytic phosphoglycerate kinase (*PGK1*) or alcohol dehydrogenase II (*ADH2*) promoters, respectively. *ADH2* is a strong promoter inducible in the absence or at low concentrations of glucose, while *PGK1* is a constitutive promoter. However, no significant difference was observed for *XYN2* expression by Y294 [ADH2-XYN] and Y294 [PGK1-XYN] strains. After 80 h of cultivation in a defined medium (Verduyn et al., 1992) containing 20 g L^−1^ glucose, specific xylanase production levels were 3.2 and 2.6 mg (g biomass)^−1^, respectively. The fermentation parameters of Y294 [PGK1-XYN] and Y294 [ADH2-XYN] were compared with those of the reference strains. In all Y294 [PGK1-XYN] and Y294 [ADH2-XYN] cultivations, a reduction in yeast biomass, ethanol, and glycerol yields were observed as well as specific consumption and production rates of glucose and ethanol, compared with the reference strains. Therefore, the expression of XYN2 from either *PGK1* or *ADH2* promoters resulted in a significant metabolic burden on the host metabolism.

These findings might explain the results obtained by Mert et al. (2016), who found lower biomass production in the engineered strain Y294 [YMXI] during cultivation on xylan than in xylose as sole carbohydrate source. It is likely that the metabolic burden associated with the expression of xylanolytic enzymes impacted xylose isomerase activity. Unfortunately, xylanase activity assays during cultivation on xylan were not reported (Mert et al., 2016). The influence of *ADH2* and *PGK1* on xylanase expression was also examined by (Nuyens et al., 2001). They also used two 2-µm yeast plasmids named pFN3 and pFN4 to insert endoxylanase (*xynA*) of *B. pumilus* PLS into *S. cerevisiae* Y294 strain under the control of these two different promoters. The two engineered yeast strains did not exhibit any xylanase activities until the gene encoding uracil phosphoribosyl transferase (*FUR1*) was disrupted. This step ensured auto-selection of the *URA3*-bearing expression plasmid in a rich growth medium since mutants by *FUR1* disruption allow the growth of the recombinant yeasts in a complex medium without the risk of losing the plasmid (La Grange et al., 1996). However, unlike the work of (Görgens et al., 2001), Y291 [pFN3 *fur1:LEU2*], in which the xylanase was under the control of the *ADH2* promoter, exhibited better xylanase activity (and presumably, expression) in the culture supernatant than Y291 [pFN4 *fur1::LEU2*], specifically 8.5 nkat ml^−1^ and 4.5 nkat ml^−1^, respectively.

Recently Niu et al. (2019), reported ethanol production from an efficient xylose-utilizing strain, BSPX042, expressing a xylose isomerase gene derived from a bovine rumen metagenomic study (Ru-*xylA*), cloned in an episomal plasmid (pJXIH-PC, *URA3* as a select marker) carrying the β-xylosidase from *Penicillium oxalicum* (*xyl3A*) and the signal peptide fragment *INU* from *Kluyveromyces* sp. The recombinant strain, BSGIBX, cultivated in a selective synthetic complete medium supplemented with 20 g L^−1^ XOS, immediately converted XOS into xylobiose and xylotriose after inoculation. The highest ethanol concentration, approximately 4,37 g L^−1^, was reached at 36 h. When the XOS were pretreated with xylanase, the ethanol concentration reached approximately 9 g L^−1^. Another important study involving the use of the XI pathway and xylanases is the work of (Tabañag et al., 2018). They expressed five different hemicellulases: endoxylanase (*XYNII*), β-xylosidase (*Bxl1*), acetylxylan esterase (*Axe1*), α-D-glucuronidase (*Glr1*) and α-L-arabinofuranosidase (*Abf1*), all from *T. reesei*, bound to the cell surface of a XI-expressing *S. cerevisiae* strain. Since hemicellulose is a complex structure that requires a consortium of enzymes to break it down completely, the authors explored accessory enzymes to make the main-chain more accessible to main-chain cleaving hemicellulases. The recombinant strain grew on xylan substrates as their sole carbon source and achieved an ethanol titer of 0.96 g L^−1^ after 160 h of cultivation.

In the context of a lignocellulosic biorefinery, in order to make full use of cellulose and hemicellulose to produce ethanol, Lee et al. (2007) investigated constitutive co-expression of endoxylanase (*xynA*) from *Bacillus* spp. and endoglucanase (*egl6*) from *Trichoderma* spp. in *S. cerevisiae* SEY2102 strain. The expression levels of endoxylanase and endoglucanase were investigated during aerobic cultivation on YPD medium. Although fermentative parameters were not investigated, 5.6 U mL^−1^ of endoxylanase was secreted into the extracellular medium, and 1.96 U mL^−1^ was intracellular after 48 h cultivation. However, these findings are still far from achieving the goal of ethanol bioconversion from cellulosic biomass. The most promising strategy for converting cellulosic biomass to ethanol in yeast is certainly the concerted heterologous expression of all main types of hemicellulases and cellulases enzymes to maximize their synergies and improve ethanol production (Nevoigt, 2008).

Similarly to the previous report, Saitoh et al. (2011) have reported the expression of β-xylosidase and β-glucosidase from *T. reesei* on the yeast cell surface based on α-agglutinin engineering system, obtaining the engineered industrial *S. cerevisiae* strain OC2-AXYL2-ABGL2-Xyl2 which also contains the oxidoreductase pathway for xylose consumption. The highest ethanol concentration, 12.5 g L^−1^, was observed after 48 h in YPKX medium (40 g L^−1^ KC-flock and 40 g L^−1^ xylan from Birchwood) containing 30 g L^−1^ cellulose. The ethanol yield was 0.52 g (g sugar consumed)^−1^. Another example of the coexpression in *S. cerevisiae* of cellulase and hemicellulose enzymes, including an endoxylanase, xylosidase, and glucosidase was reported by (Sakamoto et al., 2012). These authors expressed endoxylanase from *T. reesei*, β-xylosidase from *Aspergillus oryzae*, and β-glucosidase from *A. aculeatus* anchored on the surface cell of the laboratory xylose-assimilating *S. cerevisiae* MN8140/XBX. Therefore, the recombinant strain, MN81/XBXX, expressed XR and XDH from *S. stipitis*, xylulokinase from *S. cerevisiae*, in addition to xylanases, and cellulase enzymes. The strain was reported to ferment cellulose and hemicellulose giving a high ethanol yield, 0.32 g g^−1^, and concentration of 8.2 g L^−1^ after 72 h, from rice straw under oxygen-limited conditions and initial cell concentration of 100 g of wet cells L^−1^. When 0.2 g L^−1^ of a commercial hemicellulase was added at the medium, the recombinant strain reached 10.3 g L^−1^ ethanol after 72 h, and the ethanol yield was 0.41 g g^−1^. The addition of the commercial hemicellulase allows complete hydrolysis of xylobiose after 72 h of fermentation, which in turn increased xylose content in the medium. Although the depletion of all xylobiose, after 72 h of fermentation 2.2 g L^−1^ xylose remained in the fermentation medium (Sakamoto et al., 2012).

Lastly, Xiao et al. (2019) also reported the co-expression of both cellulase and xylanase enzymes in *S. cerevisiae* (unfortunately, the authors did not specify what cellulase or xylanase was used in their work). As reported herein, there is a synergistic action between cellulase and xylanase during lignocellulosic hydrolysate (Zhang et al., 2012; Wang et al., 2018; Xiao et al., 2019). In the presence of such synergies, pretreated lignocellulosic substrate degradation is more efficient because, since XOS can inhibit cellulase activity, the co-expression strategy would reduce the cellulase activity inhibitors (Zhang et al., 2012; Wang et al., 2018). Inside the concept of synergistic effect, it is worth noting that β-glucosidase did not affect xylanase activity as demonstrated by Chen et al. (2019b). The recombinant strains of Xiao et al. (2019), *INVSc1*-CBH-CA and *INVSc1*-CBH-TS, were cultivated using partly delignified corn stover (PDCS) producing 1.66 g L^−1^ and 1.90 g L^−1^ of ethanol after 120 h cultivation, respectively. This was approximately 4 times higher than the control (a strain that expressed a single cellulase or xylanase). Although the ethanol production did not exceed that of other published works (Katahira et al., 2004; Fujii et al., 2011; Niu et al., 2019) the effective synergistic effect of those enzymes could improve the saccharification of lignocellulose and increase the ethanol yield during fermentation by *S. cerevisiae*.

It is important to note that most of the investigations using engineered xylan-consuming *S. cerevisiae* cells have been carried out using laboratory strains, except by the work of (Saitoh et al., 2011). However, based on the industrial conditions for ethanol production, i.e., lignocellulosic inhibitors (Almeida et al., 2007), high osmolarity, and low pH, industrial host backgrounds would present more advantages as compared to laboratory strains (Della-Bianca and Gombert, 2013; Cola et al., 2020).

## Ethanol Production From Intracellular XOS Utilization in *S. cerevisiae* Strains

Economic bioethanol production from lignocellulose requires complete and rapid conversion of both cellulose and hemicellulose on an industrial scale (Li et al., 2015; Mert et al., 2016). This generally includes the pretreatment of lignocellulosic biomass to increase enzyme accessibility, which improves the amount of fermentable sugars from the enzymatic digestion for biomass-to-bioethanol microbial conversion, (Palmqvist and Hahn-Hägerdal, 2000a; Katahira et al., 2004; Lynd et al., 2008). Ultimately, engineered *S. cerevisiae* expressing XOS-transporters and producing active xylanolytic enzymes for the intracellular depolymerization of XOS to xylose are important for reducing the xylanases inhibition by its end products and for tackling the issues of microbial contamination in industrial conditions, as well as for taking full advantage of all of the sugars in lignocellulosic hydrolysate (Fujii et al., 2011; Niu et al., 2019). There is a large amount of data on the expression of xylanolytic enzymes in *S. cerevisiae* strains, however, few of them report the expression of intracellular XOS hydrolysis system in this yeast (Li et al., 2015). Besides the reduction of xylanases inhibition by its end products, XOS internalization represents an additional advantage over extracellular hydrolysis. The engineered *S. cerevisiae* would grow faster than other contaminant microorganisms in the fermentation tank.

Neither XOS transporters in *S. cerevisiae* nor expression of heterologous XOS-transporters expression have been reported in the works mentioned above. Li et al. (2015) reported the first engineered *S. cerevisiae* strain able to consume XOS intracellularly following uptake by an oligosaccharide-transporter. The recombinant strain expressed two β-xylosidases, GH43-2, and GH23-7, and one transporter, CDT-2, from *Neurospora crassa* as well as XR/XDH from *S. stipitis* to ensure the internal breakdown of XOS into ethanol (SR8U) (Kim et al., 2013b; Li et al., 2015). The expression of both β-xylosidases was essential for converting XOS into xylose as the XR acted as an XOS reductase, producing xylosyl-xylitol as a potential dead-end product. Although GH43-7 had weak β-xylosidase activity, it rapidly hydrolyzed xylosyl-xylitol into xylose and xylitol. Anaerobic fermentation with this strain, expressing CDT-2, GH43-2, and GH43-7 in an optimized minimum medium (oMM) containing 4% xylose and 3% XOS, produced more than 30 g L^−1^ of ethanol in 72 h of cultivation, after supplying an additional 50 g L^−1^ xylose at hour 48 (Li et al., 2015). The authors also performed a co-fermentation of sucrose plus XOS with the strain SR8U carrying the plasmid pXD8.7. According to their report, the recombinant strain could increase 3 g L^−1^ of ethanol concentration comparing cultivations performed in oMM media containing approximately 60 g L^−1^ sucrose (control cultivation) and the media containing approximately 60 g L^−1^ sucrose and 10 g L^−1^ XOS of which 4.2 g L^−1^ represent xylobiose and 2.3 g L^−1^ xylotriose (Li et al., 2015).

Although Fujii et al. (2011) did not have express a specific XOS transporter, their recombinant strain could hydrolyze xylobiose and xylotriose to xylose intracellularly. They have reported an XOS-fermenting yeast strain, D-XSD/XKXDHXR, that was constructed by expression of intracellular *T. reesei* β-xylosidase in a xylose-utilizing *S. cerevisiae* D452-2 strain expressing oxidoreductase pathway from *S. stipitis*. The recombinant strain D-XSD/XKXDHXR produced 4.2 g L^−1^ of ethanol from 10.8 g L^−1^ of xylobiose and 4.1 g L^−1^ of xylotriose after 168 h of fermentation in SCX medium under anaerobic conditions. The group reported xylose accumulation in the fermentative broth, suggesting that xylose uptake was a rate-limiting step, leading to a long XOS fermentation time. The authors claimed that the extracellular xylose accumulation implies that the cell exported the excess of intracellular xylose since no extracellular β-xylosidase activity was detected and the XOS hydrolysis occurred in the intracellular environment. These data suggest xylobiose and xylotriose were transported inside the cell using native transporters of the related *S. cerevisiae* strain, probably by saccharide transporters.

As evident from Table 2, chromosomal integrative approaches have not been widely applied to construct the XOS-utilizing *S. cerevisiae* strain. Although laborious, this approach represents the only feasible strategy for engineering genetically stable yeast strains without a select marker, for industrial applications (Nevoigt, 2008; Fang et al., 2017). Fang et al. (2017) reported a promising approach to obtain yeast with a chromosomal multicopy expression of a *Bacillus* sp. xylanase (*xynHB*) in *S. cerevisiae* strain A13. rDNA-mediated integration was used in their work, providing stable expression over 1,011 generations of cultivation, and higher copy numbers of the target gene in the chromosome than from integrating plasmids, i.e., 13.64 copies of *xynHB* gene were found in the A13 genome. Yeast genome contains around 100 rDNA repetitive units which provide ideal homologous recombination sites for the target gene. It is worth noting that gene stability is only observed when the integrated plasmid is smaller than the size of the rDNA unit (9.1 kb) (Lopes et al., 1996). The A13 strain lacked the enzymes required to form xylulose from xylose, therefore ethanol production was not the goal of Fang’s research.

## Concluding Remarks and Prospects

Although xylan-degrading enzyme systems have been studied extensively, there are much more missing points to connect than cellulose-degrading enzyme systems, probably because the structure of xylan is more complex and varies from plant to plant. However, the xylanolytic enzyme system deserves the same attention as the cellulolytic systems because their biotechnological potential is equally important. *S. cerevisiae* wild type strains are not suitable for producing bioethanol, even from a lignocellulosic hydrolysate with minimized production of inhibitors and high concentrations of hemicellulose/cellulose-derived oligosaccharides. A xylo-oligosaccharide-assimilating pathway has been demonstrated to be effective to generate *S. cerevisiae* strains able to convert polysaccharides into monomers. The effective intracellular hydrolysis of XOS has been demonstrated, however, the development of a strain capable of transporting large molecules of XOS is a crucial challenge. Screening for non-glucose specific transporters, such as xylose and xylo-oligomers specific transporters, and intracellular endoxylanases might advance strain improvement for efficient biomass conversion.

Significant improvements towards ethanol production from hemicellulose have been achieved in recent years, as the synergistic effect of overexpressing a combination of β-xylosidases, xylanases, and cellulases have been established (La Grange et al., 2000; Chen et al., 2019b; Xiao et al., 2019); however, there are potential limitations for efficient ethanol production from xylan by engineered *S. cerevisiae*, for example, β-Xylosidases with lower inhibition by products are needed for future *S. cerevisiae* engineering in order to achieve the complete conversion of xylobiose or xylotriose into xylose (Niu et al., 2019). In addition, it needs to be recognized that xylan in hemicellulose is typically branched and decorated, requiring accessory enzymes for their removal. Nevertheless, improvements in the technology to engineer and evolve *S. cerevisiae*, together with our current state of knowledge suggest that there is a high potential for the application of xylanolytic enzymes to obtain mono- and oligosaccharides from pretreated lignocellulose followed by fermentation into ethanol, since the cost of such sugars has historically been far too high to attract industrial interest.

Concerning the actual large-scale bioethanol production process from sugar- and starch-containing feedstock, hemicellulosic-derived bioethanol is still in its infancy due to low ethanol yield achieved by the abovementioned engineered *S. cerevisiae* strains. However, it is worth mentioning that lignocellulosic hydrolysate contains a mix of carbon sources, such as cello-oligosaccharides, XOS, and monomers as glucose, and xylose. Naturally, *S. cerevisiae* can consume some mono- and disaccharides, such as D-glucose, D-galactose, D-fructose, D-mannose, maltose, sucrose, and trehalose (Lagunas 1993), which do not represent the totality of the sugar derived from lignocellulosic hydrolysate. It is well known in the scientific literature that economically feasible bioethanol production might include the use of all sugars available in the lignocellulosic biomass. Thereby, despite the poor fermentation performance of XOS-utilizing *S. cerevisiae* strains as compared to glucose/sucrose fermentations, the simultaneous co-fermentation of lignocellulosic-derived sugars may result in higher ethanol titers and will maximize the use of the carbon sources available in lignocellulosic feedstock.

## References

[B1] Almeida J. R. Modig T. Petersson A. Hähn-Hägerdal B. Lidén G. Gorwa-Grauslund M. F. (2007). Increased Tolerance and Conversion of Inhibitors in Lignocellulosic Hydrolysates by *Saccharomyces Cerevisiae* . J. Chem. Technol. Biotechnol. 82, 340–349. 10.1002/jctb.1676

[B2] Amorim H. V. Lopes M. L. de Castro Oliveira J. V. Buckeridge M. S. Goldman G. H. (2011). Scientific Challenges of Bioethanol Production in Brazil. Appl. Microbiol. Biotechnol. 91, 1267–1275. 10.1007/s00253-011-3437-6 21735264

[B3] Banerjee G. Car S. Scott-Craig J. S. Borrusch M. S. Walton J. D. (2010). Rapid Optimization of Enzyme Mixtures for Deconstruction of Diverse Pretreatment/biomass Feedstock Combinations. Biotechnol. Biofuels 3, 1. 10.1186/1754-6834-3-22 20939889 PMC2964541

[B4] Beg Q. K. Kapoor M. Mahajan L. Hoondal G. S. (2001). Microbial Xylanases and Their Industrial Applications: A Review. Appl. Microbiol. Biotechnol. 56, 326–338. 10.1007/s002530100704 11548999

[B5] Bhardwaj N. Kumar B. Verma P. (2019). A Detailed Overview of Xylanases: an Emerging Biomolecule for Current and Future Prospective. Bioresour. Bioproc. 6, 1–40. 10.1186/s40643-019-0276-2

[B6] Biely P. (1985). Microbial Xylanolytic Systems. Trends Biotechnol. 3, 286–290. 10.1016/0167-7799(85)90004-6

[B7] Biely P. Petráková E. (1984). Novel Inducers of the Xylan-Degrading Enzyme System of Cryptococcus Albidus. J. Bacteriol. 160, 408–412. 10.1128/jb.160.1.408-412.1984 6434523 PMC214733

[B8] Biely P. Singh S. Puchart V. (2016). Towards Enzymatic Breakdown of Complex Plant Xylan Structures: State of the Art. Biotechnol. Adv. 34, 1260–1274. 10.1016/j.biotechadv.2016.09.001 27620948

[B9] Borgström C. Wasserstrom L. Almqvist H. Broberg K. Klein B. Noack S. (2019). Identification of Modifications Procuring Growth on Xylose in Recombinant *Saccharomyces cerevisiae* Strains Carrying the Weimberg Pathway. Metab. Eng. 55, 1–11. 10.1016/j.ymben.2019.05.010 31150803

[B10] Bracher J. M. Martinez-Rodriguez O. A. Dekker W. J. C. Verhoeven M. D. Van Maris A. J. A. Pronk J. T. (2019). Reassessment of Requirements for Anaerobic Xylose Fermentation by Engineered, Non-evolved *Saccharomyces cerevisiae* Strains. FEMS Yeast Res. 19, 1–15. 10.1093/femsyr/foy104 PMC6240133 30252062

[B11] Brenelli L. B. Figueiredo F. L. Damasio A. Franco T. T. Rabelo S. C. (2020). An Integrated Approach to Obtain Xylo-Oligosaccharides from Sugarcane Straw: From Lab to Pilot Scale. Bioresour. Tech. 313, 123637. 10.1016/J.BIORTECH.2020.123637 32535521

[B12] Cabrini K. T. Gallo C. R. (1999). Identificação De Leveduras No Processo De Fermentação Alcoólica Em Usina Do Estado De São Paulo, Brasil. Sci. Agric. (Piracicaba, Braz. 56, 207–216. 10.1590/S0103-90161999000100028

[B13] Chakdar H. Kumar M. Pandiyan K. Singh A. Nanjappan K. Kashyap P. L. (2016). Bacterial Xylanases: Biology to Biotechnology. 3 Biotech. 6, 1–15. 10.1007/s13205-016-0457-z PMC4929084 28330222

[B14] Chandra Raj K. Chandra T. S. (1995). A Cellulase-free Xylanase from Alkali-tolerantAspergillus Fischeri Fxn1. Biotechnol. Lett. 17, 309–314. 10.1007/BF01190644

[B15] Chávez R. Schachter K. Navarro C. Peirano A. Aguirre C. Bull P. (2002). Differences in Expression of Two Endoxylanase Genes (xynA and xynB) from Penicillium purpurogenum. Gene 293, 161–168. 10.1016/S0378-1119(02)00720-5 12137954

[B16] Chen C.-H. Yao J.-Y. Yang B. Lee H.-L. Yuan S.-F. Hsieh H.-Y. (2019b). Engineer Multi-Functional Cellulase/xylanase/β-Glucosidase with Improved Efficacy to Degrade rice Straw. Bioresour. Tech. Rep. 5, 170–177. 10.1016/j.biteb.2019.01.008

[B17] Chen P. You Q. Li X. Chang Q. Zhang Y. Zheng B. (2019a). Polysaccharide Fractions from Fortunella Margarita Affect Proliferation of Bifidobacterium Adolescentis ATCC 15703 and Undergo Structural Changes Following Fermentation. Int. J. Biol. Macromolecules 123, 1070–1078. 10.1016/J.IJBIOMAC.2018.11.163 30465831

[B18] Christakopoulos P. Mamma D. Nerinckx W. Kekos D. Macris B. Claeyssens M. (1996a). Production and Partial Characterization of Xylanase from Fusarium Oxysporum. Bioresour. Tech. 58, 115–119. 10.1016/S0960-8524(96)00091-0

[B19] Christakopoulos P. Nerinckx W. Kekos D. Macris B. Claeyssens M. (1996b). Purification and Characterization of Two Low Molecular Mass Alkaline Xylanases from Fusarium Oxysporum F3. J. Biotechnol. 51, 181–189. 10.1016/0168-1656(96)01619-7 8987884

[B20] Cola P. Procópio D. P. Alves A. T. d. C. Carnevalli L. R. Sampaio I. V. da Costa B. L. V. (2020). Differential Effects of Major Inhibitory Compounds from Sugarcane-Based Lignocellulosic Hydrolysates on the Physiology of Yeast Strains and Lactic Acid Bacteria. Biotechnol. Lett. 42, 571–582. 10.1007/s10529-020-02803-6 31974646

[B21] Collins T. Gerday C. Feller G. (2005). Xylanases, Xylanase Families and Extremophilic Xylanases. FEMS Microbiol. Rev. 29, 3–23. 10.1016/j.femsre.2004.06.005 15652973

[B22] Collins T. Hoyoux A. Dutron A. Georis J. Genot B. Dauvrin T. (2006). Use of Glycoside Hydrolase Family 8 Xylanases in Baking. J. Cereal Sci. 43, 79–84. 10.1016/j.jcs.2005.08.002

[B23] Crous J. M. Pretorius I. S. Van Zyl W. H. (1995). Cloning and Expression of an Aspergillus Kawachii Endo-1,4-β-Xylanase Gene in *Saccharomyces cerevisiae* . Curr. Genet. 28, 467–473. 10.1007/BF00310817 8575021

[B24] Crous J. M. Pretorius I. S. Van Zyl W. H. (1996). Cloning and Expression of the α- L -arabinofuranosidase Gene ( ABF 2) of Aspergillus niger in *Saccharomyces cerevisiae* . Appl. Microbiol. Biotechnol. 46, 256–260. 10.1007/s002530050813 8933843

[B25] Dahlman O. Jacobs A. Sjöberg J. (2003). Molecular Properties of Hemicelluloses Located in the Surface and Inner Layers of Hardwood and Softwood Pulps. Cellulose 10, 325–334. Available at: https://link.springer.com/content/pdf/10.1023/A:1027316926308.pdf (Accessed September 7, 2020). 10.1023/a:1027316926308

[B26] de Almeida M. A. Colombo R. (2021). Production Chain of First-Generation Sugarcane Bioethanol: Characterization and Value-Added Application of Wastes. Bioenerg. Res. 1-16. 10.1007/s12155-021-10301-4

[B27] de Vries R. P. Riley R. Wiebenga A. Aguilar-Osorio G. Amillis S. Uchima C. A. (20172017). Comparative Genomics Reveals High Biological Diversity and Specific Adaptations in the Industrially and Medically Important Fungal Genus Aspergillus. Genome Biol. 18, 28–45. 10.1186/S13059-017-1151-0 PMC5307856 28196534

[B28] Della-Bianca B. E. de Hulster E. Pronk J. T. van Maris A. J. A. Gombert A. K. (2014). Physiology of the Fuel Ethanol strain *Saccharomyces cerevisiae* PE-2 at Low pH Indicates a Context-dependent Performance Relevant for Industrial Applications. FEMS Yeast Res. 14, 1196–1205. 10.1111/1567-1364.12217 25263709

[B29] Della-Bianca B. E. Gombert A. K. (2013). Stress Tolerance and Growth Physiology of Yeast Strains from the Brazilian Fuel Ethanol Industry. Antonie van Leeuwenhoek 104, 1083–1095. 10.1007/s10482-013-0030-2 24062068

[B30] Delmas S. Pullan S. T. Gaddipati S. Kokolski M. Malla S. Blythe M. J. (2012). Uncovering the Genome-wide Transcriptional Responses of the Filamentous Fungus Aspergillus niger to Lignocellulose Using RNA Sequencing. PLoS Genet. 8, e1002875–13. 10.1371/JOURNAL.PGEN.1002875 22912594 PMC3415456

[B31] Donald K. A. G. Carle A. Gibbs M. D. Bergquist P. L. (1994). Production of a Bacterial Thermophilic Xylanase in *Saccharomyces Cerevisiae* . Appl. Microbiol. Biotechnol. 42, 309–312. 10.1007/BF00902734

[B32] dos Reis T. F. de Lima P. B. A. Parachin N. S. Mingossi F. B. de Castro Oliveira J. V. Ries L. N. A. (2016). Identification and Characterization of Putative Xylose and Cellobiose Transporters in Aspergillus nidulans. Biotechnol. Biofuels 9, 204. 10.1186/s13068-016-0611-1 27708711 PMC5037631

[B33] Dos Santos L. V. Carazzolle M. F. Nagamatsu S. T. Sampaio N. M. V. Almeida L. D. Pirolla R. A. S. (2016). Unraveling the Genetic Basis of Xylose Consumption in Engineered *Saccharomyces cerevisiae* Strains. Sci. Rep. 6, 38676. 10.1038/srep38676 28000736 PMC5175268

[B34] Eliasson A. Christensson C. Wahlbom C. F. Hahn-Hägerdal B. (2000). Anaerobic Xylose Fermentation by Recombinant *Saccharomyces cerevisiae* Carrying XYL1 , XYL2 , and XKS1 in Mineral Medium Chemostat Cultures. Appl. Environ. Microbiol. 66, 3381–3386. 10.1128/AEM.66.8.3381-3386.2000 10919795 PMC92159

[B35] Fang C. Wang Q. Selvaraj J. N. Zhou Y. Ma L. Zhang G. (2017). High Copy and Stable Expression of the Xylanase XynHB in *Saccharomyces cerevisiae* by rDNA-Mediated Integration. Sci. Rep. 7, 1–9. 10.1038/s41598-017-08647-x 28821784 PMC5562786

[B36] Fernandes Pereira P. H. Cornelis H. Voorwald J. Odila M. Cioffi H. Mulinari D. R. (2011). Sugarcane Bagasse Pulping and Bleaching: Thermal and Chemical Characterization. BioResources 6, 2471–2482. Available at: https://bioresources.cnr.ncsu.edu/wp-content/uploads/2016/06/BioRes_06_3_2471_FernandesP_VCMDP_SCB_Pulp_Bleach_Therm_Chem_Char_987.pdf 10.15376/biores.6.1.867-878

[B37] Frommer W. B. Ninnemann O. (1995). Heterologous Expression of Genes in Bacterial, Fungal, Animal, and Plant Cells. Annu. Rev. Plant Physiol. Plant Mol. Biol. 46, 419–444. 10.1111/1751-7915.12872

[B38] Fujii T. Yu G. Matsushika A. Kurita A. Yano S. Murakami K. (2011). Ethanol Production from Xylo-Oligosaccharides by Xylose-Fermenting *Saccharomyces cerevisiae* Expressing β-Xylosidase. Biosci. Biotechnol. Biochem. 75, 1140–1146. 10.1271/bbb.110043 21670522

[B39] Fujita Y. Katahira S. Ueda M. Tanaka A. Okada H. Morikawa Y. (2002). Construction of Whole-Cell Biocatalyst for Xylan Degradation through Cell-Surface Xylanase Display in *Saccharomyces cerevisiae* . J. Mol. Catal. B: Enzymatic 17, 189–195. 10.1016/S1381-1177(02)00027-9

[B40] Galbe M. Zacchi G. (2002). A Review of the Production of Ethanol from Softwood. Appl. Microbiol. Biotechnol. 59, 618–628. 10.1007/s00253-002-1058-9 12226717

[B41] Gárdonyi M. Hahn-Hägerdal B. (2003). The Streptomyces Rubiginosus Xylose Isomerase Is Misfolded when Expressed in *Saccharomyces cerevisiae* . Enzyme Microb. Technol. 32, 252–259. 10.1016/S0141-0229(02)00285-5

[B42] Gírio F. M. Fonseca C. Carvalheiro F. Duarte L. C. Marques S. Bogel-Łukasik R. (2010). Hemicelluloses for Fuel Ethanol: A Review. Bioresour. Tech. 101, 4775–4800. 10.1016/j.biortech.2010.01.088 20171088

[B43] Görgens J. F. van Zyl W. H. Knoetze J. H. Hahn-Hägerdal B. (2001). The Metabolic burden of thePGK1andADH2promoter Systems for Heterologous Xylanase Production by *Saccharomyces Cerevisiae* in Defined Medium. Biotechnol. Bioeng. 73, 238–245. 10.1002/bit.1056 11257606

[B44] Grange D. C. L. Pretorius I. S. Van Zyl W. H. (1997). Cloning of the Bacillus Pumilus β-xylosidase Gene ( xynB ) and its Expression in *Saccharomyces cerevisiae* . Appl. Microbiol. Biotechnol. 47, 262–266. 10.1007/s002530050924 9114518

[B45] Gueguen Y. Chemardin P. Labrot P. Arnaud A. Galzy P. (1997). Purification and Characterization of an Intracellular β‐glucosidase from a New Strain of Leuconostoc Mesenteroides Isolated from Cassava. J. Appl. Microbiol. 82, 469–476. 10.1046/j.1365-2672.1997.00136.x

[B46] Gupta A. Verma J. P. (2015). Sustainable Bio-Ethanol Production from Agro-Residues: A Review. Renew. Sust. Energ. Rev. 41, 550–567. 10.1016/J.RSER.2014.08.032

[B47] Harner N. K. Wen X. Bajwa P. K. Austin G. D. Ho C.-Y. Habash M. B. (2015). Genetic Improvement of Native Xylose-Fermenting Yeasts for Ethanol Production. J. Ind. Microbiol. Biotechnol. 42, 1–20. 10.1007/s10295-014-1535-z 25404205

[B49] Honda Y. Kitaoka M. (2004). A Family 8 Glycoside Hydrolase from Bacillus Halodurans C-125 (BH2105) Is a Reducing End Xylose-Releasing Exo-Oligoxylanase. J. Biol. Chem. 279, 55097–55103. 10.1074/jbc.M409832200 15491996

[B50] Hou J. Tyo K. E. J. Liu Z. Petranovic D. Nielsen J. (2012). Metabolic Engineering of Recombinant Protein Secretion by *Saccharomyces cerevisiae* . FEMS Yeast Res. 12, 491–510. 10.1111/J.1567-1364.2012.00810.X 22533807

[B51] Jacobsen S. E. Wyman C. E. (2000). Cellulose and Hemicellulose Hydrolysis Models for Application to Current and Novel Pretreatment Processes. Appl. Biochem. Biotechnol. 84, 81–96. 10.1385/ABAB:84-86:1-9:81 10849781

[B52] Jeffries T. W. (1983). “Utilization of Xylose by Bacteria, Yeasts, and Fungi,” in Pentoses and Lignin (Berlin/Heidelberg: Springer-Verlag), 1–32. 10.1007/BFb0009101 6437152

[B53] Johnsen U. Dambeck M. Zaiss H. Fuhrer T. Soppa J. Sauer U. (2009). D-xylose Degradation Pathway in the Halophilic Archaeon Haloferax Volcanii. J. Biol. Chem. 284, 27290–27303. 10.1074/jbc.M109.003814 19584053 PMC2785657

[B54] Jørgensen H. Kristensen J. B. Felby C. (2007). Enzymatic Conversion of Lignocellulose into Fermentable Sugars: Challenges and Opportunities. Biofuels, Bioprod. Bioref. 1, 119–134. 10.1002/bbb.4

[B55] Katahira S. Fujita Y. Mizuike A. Fukuda H. Kondo A. (2004). Construction of a Xylan-Fermenting Yeast Strain through Codisplay of Xylanolytic Enzymes on the Surface of Xylose-Utilizing *Saccharomyces cerevisiae* Cells. Appl. Environ. Microbiol. 70, 5407–5414. 10.1128/AEM.70.9.5407-5414.2004 15345427 PMC520881

[B56] Kim J.-H. Kim B.-W. Yoon K.-H. Nam S.-W. (2000). Expression of Bacillus Sp. β-xylosidase Gene (xylB) in *Saccharomyces cerevisiae* . Biotechnol. Lett. 22, 1025–1029. 10.1023/a:1005661909095

[B57] Kim M.-J. Kim B.-H. Nam S.-W. Choi E.-S. Shin D.-H. Cho H.-Y. (2013a). Efficient Secretory Expression of Recombinant Endoxylanase from Bacillus Sp. HY-20 in *Saccharomyces cerevisiae* . J. Life Sci. 23, 863–868. 10.5352/jls.2013.23.7.863

[B58] Kim S. R. Ha S.-J. Wei N. Oh E. J. Jin Y.-S. (2012). Simultaneous Co-fermentation of Mixed Sugars: A Promising Strategy for Producing Cellulosic Ethanol. Trends Biotechnol. 30, 274–282. 10.1016/j.tibtech.2012.01.005 22356718

[B59] Kim S. R. Skerker J. M. Kang W. Lesmana A. Wei N. Arkin A. P. (2013b). Rational and Evolutionary Engineering Approaches Uncover a Small Set of Genetic Changes Efficient for Rapid Xylose Fermentation in *Saccharomyces cerevisiae* . PLoS One 8, e57048–13. 10.1371/journal.pone.0057048 23468911 PMC3582614

[B60] Kulkarni N. Shendye A. Rao M. (1999). Molecular and Biotechnological Aspects of Xylanases. FEMS Microbiol. Rev. 23, 411–456. 10.1111/j.1574-6976.1999.tb00407.x 10422261

[B61] Kuyper M. Hartog M. Toirkens M. Almering M. Winkler A. Vandijken J. (2005). Metabolic Engineering of a Xylose-Isomerase-Expressing Strain for Rapid Anaerobic Xylose Fermentation. FEMS Yeast Res. 5, 399–409. 10.1016/j.femsyr.2004.09.010 15691745

[B62] Kwak S. Jin Y.-S. (2017). Production of Fuels and Chemicals from Xylose by Engineered *Saccharomyces cerevisiae*: A Review and Perspective. Microb. Cel. Fact. 16, 1–15. 10.1186/s12934-017-0694-9 PMC5425999 28494761

[B63] La Grange D. C. Claeyssens M. Pretorius I. S. Van Zyl W. H. (2000). Coexpression of the Bacillus Pumilus β-xylosidase ( xynB ) Gene with the Trichoderma Reesei β-xylanase 2 ( Xyn2 ) Gene in the Yeast *Saccharomyces cerevisiae* . Appl. Microbiol. Biotechnol. 54, 195–200. 10.1007/s002530000372 10968632

[B64] La Grange D. C. Pretorius I. S. Claeyssens M. Van Zyl W. H. (2001). Degradation of Xylan to D -Xylose by Recombinant *Saccharomyces cerevisiae* Coexpressing the Aspergillus niger β-Xylosidase ( xlnD ) and the Trichoderma Reesei Xylanase II ( Xyn2 ) Genes. Appl. Environ. Microbiol. 67, 5512–5519. 10.1128/AEM.67.12.5512-5519.2001 11722900 PMC93337

[B65] La Grange D. C. Pretorius I. S. Van Zyl W. H. (1996). Expression of a Trichoderma Reesei Beta-Xylanase Gene (XYN2) in *Saccharomyces cerevisiae* . Appl. Environ. Microbiol. 62, 1036–1044. 10.1128/aem.62.3.1036-1044.1996 8975597 PMC167867

[B66] Lagunas R. (1993). Sugar Transport in *Saccharomyces Cerevisiae* . FEMS Microbiol. Rev. 104, 229–242. 10.1111/J.1574-6968.1993.TB05869.X 8318258

[B67] Lee J. H. Lim M. Y. Kim M. J. Heo S. Y. Seo J. H. Kim Y. H. (2007). Constitutive Coexpression of Bacillus Endoxylanase and Trichoderma Endoglucanase Genes in *Saccharomyces cerevisiae* . J. Microbiol. Biotechnol. 17, 2076–2080. Available at: http://www.jmb.or.kr/submission/Journal/017/JMB017-12-24.pdf . 18167459

[B68] Lee J. H. Heo S.-Y. Lee J.-W. Yoon K.-H. Kim Y.-H. Nam S.-W. (2009). Thermostability and Xylan-Hydrolyzing Property of Endoxylanase Expressed in Yeast *Saccharomyces cerevisiae* . Biotechnol. Bioproc. E 14, 639–644. 10.1007/s12257-009-0014-2

[B69] Lee S.-M. Jellison T. Alper H. S. (2015). Xylan Catabolism Is Improved by Blending Bioprospecting and Metabolic Pathway Engineering in *Saccharomyces Cerevisiae* . Biotechnol. J. 10, 575. 10.1002/biot.201400622 25651533

[B70] Li X. Chen Y. Nielsen J. (2019). Harnessing Xylose Pathways for Biofuels Production. Curr. Opin. Biotechnol. 57, 56–65. 10.1016/j.copbio.2019.01.006 30785001

[B71] Li X. L. Ljungdahl L. G. (1996). Expression of Aureobasidium Pullulans xynA in, and Secretion of the Xylanase from, *Saccharomyces cerevisiae* . Appl. Environ. Microbiol. 62, 209–213. 10.1128/aem.62.1.209-213.1996 8572698 PMC167788

[B72] Li X. Yu V. Y. Lin Y. Chomvong K. Estrela R. Park A. (2015). Expanding Xylose Metabolism in Yeast for Plant Cell wall Conversion to Biofuels. Elife 4. e05896, 10.7554/eLife.05896 PMC4338637 25647728

[B73] Li Z. Qu H. Li C. Zhou X. (2013). Direct and Efficient Xylitol Production from Xylan by *Saccharomyces cerevisiae* through Transcriptional Level and Fermentation Processing Optimizations. Bioresour. Tech. 149, 413–419. 10.1016/j.biortech.2013.09.101 24128404

[B74] Liu L. Cheng J. Chen H. Li X. Wang S. Song A. (2011). Directed Evolution of a Mesophilic Fungal Xylanase by Fusion of a Thermophilic Bacterial Carbohydrate-Binding Module. Process Biochem. 46, 395–398. 10.1016/j.procbio.2010.07.026

[B75] Liu L. Qin Y. Wang Y. Li H. Shang N. Li P. (2014). Complete Genome Sequence of Bifidobacterium Animalis RH, a Probiotic Bacterium Producing Exopolysaccharides. J. Biotechnol. 189, 86–87. 10.1016/J.JBIOTEC.2014.08.041 25242662

[B76] Lopes D. D. Rosa C. A. Hector R. E. Dien B. S. Mertens J. A. Ayub M. A. Z. (2017). Influence of Genetic Background of Engineered Xylose-Fermenting Industrial *Saccharomyces cerevisiae* Strains for Ethanol Production from Lignocellulosic Hydrolysates. J. Ind. Microbiol. Biotechnol. 44, 1575–1588. 10.1007/s10295-017-1979-z 28891041

[B77] Lopes T. S. de Wijs I. J. Steenhauer S. I. Verbakel J. Planta R. J. (1996). Factors Affecting the Mitotic Stability of High-Copy-Number Integration into the Ribosomal DNA of *Saccharomyces Cerevisiae* . Yeast 12, 467–477. 10.1002/(SICI)1097-0061 8740420

[B78] Luttig M. Pretorius I. S. Zyl W. H. v. (1997). Cloning of Two β-xylanase-encoding Genes from Aspergillus niger and Their Expression in *Saccharomyces cerevisiae* . Biotechnol. Lett. 19, 411–415. 10.1023/A:1018327623422

[B79] Lynd L. R. Laser M. S. Bransby D. Dale B. E. Davison B. Hamilton R. (2008). How Biotech Can Transform Biofuels. Nat. Biotechnol. 26, 169–172. 10.1038/nbt0208-169 18259168

[B80] Lynd L. R. Weimer P. J. van Zyl W. H. Pretorius I. S. (2002).Microbial Cellulose Utilization: Fundamentals and Biotechnology, Microbiol. Mol. Biol. Rev. 66, 506–577. 10.1128/MMBR.66.3.506-577.2002/ASSET/38C13E20-0334-4477-8327-F000A9C877DC/ASSETS/GRAPHIC/MR032001411T 12209002 PMC120791

[B81] Ma J. Ptashne M. (1987). Deletion Analysis of GAL4 Defines Two Transcriptional Activating Segments. Cell 48, 847–853. 10.1016/0092-8674(87)90081-X 3028647

[B82] Margaritis A. Bajpai P. (1982). Direct Fermentation of D -Xylose to Ethanol by *Kluyveromyces Marxianus* Strains. Appl. Environ. Microbiol. 44, 1039–1041. 10.1128/aem.44.5.1039-1041.1982 16346128 PMC242145

[B83] Margolles-Clark E. Tenkanen M. Nakari-Setälä T. Penttilä M. Seta¨la¨ S. Penttila¨* M. (1996). Cloning of Genes Encoding Alpha-L-Arabinofuranosidase and Beta-Xylosidase from Trichoderma Reesei by Expression in *Saccharomyces cerevisiae* . Appl. Environ. Microbiol. 62, 3840–3846. 10.1128/aem.62.10.3840-3846.1996 8837440 PMC168192

[B84] Mert M. J. la Grange D. C. Rose S. H. van Zyl W. H. (2016). Engineering of *Saccharomyces cerevisiae* to Utilize Xylan as a Sole Carbohydrate Source by Co-expression of an Endoxylanase, Xylosidase and a Bacterial Xylose Isomerase. J. Ind. Microbiol. Biotechnol. 43, 431–440. 10.1007/s10295-015-1727-1 26749525

[B85] Mishra C. Keskar S. Rao M. (1984). Production and Properties of Extracellular Endoxylanase from Neurospora Crassa. Appl. Environ. Microbiol. 48, 224–228. 10.1128/aem.48.1.224-228.1984 16346591 PMC240376

[B86] Mittelman K. Barkai N. (2017). The Genetic Requirements for Pentose Fermentation in Budding Yeast. G3 Genes, Genomes, Genet. 7, 1743–1752. 10.1534/g3.117.039610 PMC5473754 28404660

[B87] Moreau A. Durand S. Morosoli R. (1992). Secretion of a Cryptococcus Albidus Xylanase in *Saccharomyces cerevisiae* . Gene 116, 109–113. 10.1016/0378-1119(92)90637-5 1628837

[B88] Moreira L. R. S. Filho E. X. F. (2016). Insights into the Mechanism of Enzymatic Hydrolysis of Xylan. Appl. Microbiol. Biotechnol. 100, 5205–5214. 10.1007/s00253-016-7555-z 27112349

[B89] Najjarzadeh N. Matsakas L. Rova U. Christakopoulos P. (2020). Effect of Oligosaccharide Degree of Polymerization on the Induction of Xylan-Degrading Enzymes by Fusarium Oxysporum F. Sp. Lycopersici. Molecules 25, 5849. 10.3390/molecules25245849 PMC7764074 33322262

[B90] Nevoigt E. (2008). Progress in Metabolic Engineering of *Saccharomyces cerevisiae* . Microbiol. Mol. Biol. Rev. 72, 379–412. 10.1128/MMBR.00025-07 18772282 PMC2546860

[B91] Nielsen J. (2019). Yeast Systems Biology: Model Organism and Cell Factory. Biotechnol. J. 14, 1800421. 10.1002/biot.201800421 30925027

[B92] Nieves R. A. Ehrman C. I. Adney W. S. Elander R. T. Himmel M. E. (1997). Survey and Analysis of Commercial Cellulase Preparations Suitable for Biomass Conversion to Ethanol. World J. Microbiol. Biotechnol. 14, 301–304. 10.1023/A:1008871205580

[B93] Niu Y. Wu L. Shen Y. Zhao J. Zhang J. Yi Y. (2019). Coexpression of β-xylosidase and Xylose Isomerase in *Saccharomyces cerevisiae* Improves the Efficiency of Saccharification and Fermentation from Xylo-Oligosaccharides. Cellulose 26, 7923–7937. 10.1007/s10570-019-02650-3

[B94] Nuyens F. Van Zyl W. H. Iserentant D. Verachtert H. Michiels C. (2001). Heterologous Expression of the Bacillus Pumilus Endo-β-Xylanase ( xynA ) Gene in the Yeast *Saccharomyces cerevisiae* . Appl. Microbiol. Biotechnol. 56, 431–434. 10.1007/s002530100670 11549015

[B95] Oh E. J. Jin Y.-S. (2020). Engineering of *Saccharomyces cerevisiae* for Efficient Fermentation of Cellulose. FEMS Yeast Res. 20. 10.1093/femsyr/foz089 31917414

[B96] Palmqvist E. Hahn-Hägerdal B. (2000a). Fermentation of Lignocellulosic Hydrolysates. I: Inhibition and Detoxification. Bioresour. Technol. 187, 228–234. 10.1016/j.biortech.2015.03.129

[B97] Palmqvist E. Hahn-Hägerdal B. (2000b). Fermentation of Lignocellulosic Hydrolysates. II: Inhibitors and Mechanisms of Inhibition. Bioresour. Tech. 74, 25–33. 10.1016/S0960-8524(99)00161-3

[B98] Pan C.-X. Xu W.-Z. Akatsuka H. Negoro S. Shima Y. Urabe I. (1991). Expression of the Xylan-Degrading Genes of Bacillus Pumilus IPO in *Saccharomyces cerevisiae* . J. Ferment. Bioeng. 71, 303–308. 10.1016/0922-338X(91)90340-M

[B99] Panbangred W. Shinmyo A. Kinoshita S. Okada H. (1983). Purification and Properties of Endoxylanase Produced by bacillus Pumilus. Agric. Biol. Chem. 47, 957–963. 10.1080/00021369.1983.1086576010.1271/bbb1961.47.957

[B100] Parachin N. S. Siqueira S. de Faria F. P. Torres F. A. G. de Moraes L. M. P. (2009). Xylanases from Cryptococcus Flavus Isolate I-11: Enzymatic Profile, Isolation and Heterologous Expression of CfXYN1 in *Saccharomyces cerevisiae* . J. Mol. Catal. B: Enzymatic 59, 52–57. 10.1016/j.molcatb.2008.12.018

[B101] Patiño M. A. Ortiz J. P. Velásquez M. Stambuk B. U. (2019). D‐Xylose Consumption by nonrecombinant *Saccharomyces Cerevisiae*: A Review. Yeast 36, 3429. 10.1002/yea.3429 31254359

[B102] Pedersen M. Meyer A. S. (2010). Lignocellulose Pretreatment Severity - Relating pH to Biomatrix Opening. New Biotechnol. 27, 739–750. 10.1016/J.NBT.2010.05.003 20460178

[B103] Peng S. Cao Q. Qin Y. Li X. Liu G. Qu Y. (2017). An Aldonolactonase AltA from Penicillium oxalicum Mitigates the Inhibition of β-glucosidase during Lignocellulose Biodegradation. Appl. Microbiol. Biotechnol. 101, 3627–3636. 10.1007/s00253-017-8134-7 28161729

[B104] Pérez-Gonzalez J. A. De Graaff L. H. Visser J. Ramón D. (1996). Molecular Cloning and Expression in *Saccharomyces cerevisiae* of Two Aspergillus nidulans Xylanase Genes. Appl. Environ. Microbiol. 62, 2179–2182. 10.1128/aem.62.6.2179-2182.1996 8787417 PMC167998

[B105] Petrescu I. Lamotte-Brasseur J. Chessa J.-P. Ntarima P. Claeyssens M. Devreese B. (2000). Xylanase from the Psychrophilic Yeast Cryptococcus Adeliae. Extremophiles 4, 137–144. 10.1007/s007920070028 10879558

[B106] Polizeli M. L. T. M. Rizzatti A. C. S. Monti R. Terenzi H. F. Jorge J. A. Amorim D. S. (2005). Xylanases from Fungi: Properties and Industrial Applications. Appl. Microbiol. Biotechnol. 67, 577–591. 10.1007/s00253-005-1904-7 15944805

[B107] Qian Y. Yomano L. P. Preston J. F. Aldrich H. C. Ingram L. O. (2003). Cloning, Characterization, and Functional Expression of the Klebsiella Oxytoca Xylodextrin Utilization Operon ( xynTB ) in *Escherichia coli* . Appl. Environ. Microbiol. 69, 5957–5967. 10.1128/AEM.69.10.5957-5967.2003 14532050 PMC201249

[B108] Rennie E. A. Scheller H. V. (2014). Xylan Biosynthesis. Curr. Opin. Biotechnol. 26, 100–107. 10.1016/j.copbio.2013.11.013 24679265

[B109] Royer J. C. Nakas J. P. (1989). Xylanase Production by Trichoderma Longibrachiatum. Enzyme Microb. Tech. 11, 405–410. 10.1016/0141-0229(89)90134-8

[B110] Ryabova O. Chmil O. Sibirny A. (2003). Xylose and Cellobiose Fermentation to Ethanol by the Thermotolerant Methylotrophic Yeast. FEMS Yeast Res. 4, 157–164. 10.1016/S1567-1356(03)00146-6 14613880

[B111] Saito Y. Shigehisa A. Watanabe Y. Tsukuda N. Moriyama-Ohara K. Hara T. (2020). Multiple Transporters and Glycoside Hydrolases Are Involved in Arabinoxylan-Derived Oligosaccharide Utilization in Bifidobacterium Pseudocatenulatum. Appl. Environ. Microbiol. 86. 10.1128/AEM.01782-20/SUPPL_FILE/AEM.01782-20-S0001 PMC7688211 33036985

[B112] Saitoh S. Tanaka T. Kondo A. (2011). Co-fermentation of Cellulose/xylan Using Engineered Industrial Yeast Strain OC-2 Displaying Both β-glucosidase and β-xylosidase. Appl. Microbiol. Biotechnol. 91, 1553–1559. 10.1007/s00253-011-3357-5 21643701

[B113] Sakamoto T. Hasunuma T. Hori Y. Yamada R. Kondo A. (2012). Direct Ethanol Production from Hemicellulosic Materials of rice Straw by Use of an Engineered Yeast Strain Codisplaying Three Types of Hemicellulolytic Enzymes on the Surface of Xylose-Utilizing *Saccharomyces cerevisiae* Cells. J. Biotechnol. 158, 203–210. 10.1016/j.jbiotec.2011.06.025 21741417

[B114] Sarthy A. V. McConaughy B. L. Lobo Z. Sundstrom J. A. Furlong C. E. Hall B. D. (19872000). Expression of the *Escherichia coli* Xylose Isomerase Gene in *Saccharomyces cerevisiae* . Appl. Environ. Microbiol. 53, 1996–2000. 10.1128/aem.53.9.1996-2000.1987 PMC204047 2823706

[B115] Schuster A. Schmoll M. (2010). Biology and Biotechnology of Trichoderma. Appl. Microbiol. Biotechnol. 87, 787–799. 10.1007/s00253-010-2632-1 20461510 PMC2886115

[B116] Sekar R. Shin H. D. DiChristina T. J. (2016). Direct Conversion of Cellulose and Hemicellulose to Fermentable Sugars by a Microbially-Driven Fenton Reaction. Bioresour. Tech. 218, 1133–1139. 10.1016/j.biortech.2016.07.087 27469094

[B117] Shen L. Kohlhaas M. Enoki J. Meier R. Schönenberger B. Wohlgemuth R. (2020). A Combined Experimental and Modelling Approach for the Weimberg Pathway Optimisation. Nat. Commun. 11, 1–13. 10.1038/s41467-020-14830-y 32107375 PMC7046635

[B118] Shulami S. Zaide G. Zolotnitsky G. Langut Y. Feld G. Sonenshein A. L. (2007). A Two-Component System Regulates the Expression of an ABC Transporter for Xylo-Oligosaccharides in Geobacillus Stearothermophilus. Appl. Environ. Microbiol. 73, 874–884. 10.1128/AEM.02367-06 17142383 PMC1800775

[B119] Smiley K. L. Bolen P. L. (1982). Demonstration of D-Xylose Reductase and D-Xylitol Dehydrogenase in Pachysolen Tannophilus. Biotechnol. Lett. 4, 607–610. 10.1007/BF00127793

[B120] Stephens C. Christen B. Fuchs T. Sundaram V. Watanabe K. Jenal U. (2007). Genetic Analysis of a Novel Pathway for D -Xylose Metabolism in *Caulobacter crescentus* . J. Bacteriol. 189, 2181–2185. 10.1128/JB.01438-06 17172333 PMC1855722

[B121] Stincone A. Prigione A. Cramer T. Wamelink M. M. C. Campbell K. Cheung E. (2015). The Return of Metabolism: Biochemistry and Physiology of the Pentose Phosphate Pathway. Biol. Rev. 90, 927–963. 10.1111/brv.12140 25243985 PMC4470864

[B122] Subramaniyan S. Prema P. (2002). Biotechnology of Microbial Xylanases: Enzymology, Molecular Biology, and Application. Crit. Rev. Biotechnol. 22, 33–64. 10.1080/07388550290789450 11958335

[B123] Sun J. Wen F. Si T. Xu J.-H. Zhao H. (2012). Direct Conversion of Xylan to Ethanol by Recombinant *Saccharomyces cerevisiae* Strains Displaying an Engineered Minihemicellulosome. Appl. Environ. Microbiol. 78, 3837–3845. 10.1128/AEM.07679-11 22447594 PMC3346407

[B124] Sun Y. Cheng J. (2002). Hydrolysis of Lignocellulosic Materials for Ethanol Production: A Review. Bioresour. Tech. 83, 1–11. 10.1016/S0960-8524(01)00212-7 12058826

[B125] Sunna A. Antranikian G. (1997). Xylanolytic Enzymes from Fungi and Bacteria. Crit. Rev. Biotechnol. 17, 39–67. 10.3109/07388559709146606 9118232

[B126] Tabañag I. D. F. Chu I.-M. Wei Y.-H. Tsai S.-L. (2018). Ethanol Production from Hemicellulose by a Consortium of Different Genetically-Modified *Sacharomyces Cerevisiae* . J. Taiwan Inst. Chem. Eng. 89, 15–25. 10.1016/j.jtice.2018.04.029

[B127] Tafakori V. Torktaz I. Doostmohamadi M. Ahmadain G. (2012). Microbial Cell Surface Display; its Medical and Environmental Applications. Iran. J. Biotechnol. 10, 231–239. Available at: https://www.researchgate.net/publication/232708479_Microbial_cell_surface_display_its_medical_and_environmental_applications (Accessed January 21, 2021).

[B128] Tengborg C. Stenberg K. Galbe M. Zacchi G. Larsson S. Palmqvist E. (1998). Comparison of SO2 and H2SO4 Impregnation of Softwood Prior to Steam Pretreatment on Ethanol Production. Applied Biochemistry and Biotechnology 70-2, 3–15. 10.1007/BF02920119

[B129] Tian B. Xu Y. Cai W. Huang Q. Gao Y. Li X. (2013). Molecular Cloning and Overexpression of an Endo-β-1,4-Xylanase Gene from Aspergillus niger in Industrial *Saccharomyces cerevisiae* YS2 Strain. Appl. Biochem. Biotechnol. 170, 320–328. 10.1007/s12010-013-0173-7 23508862

[B130] Toivola A. Yarrow D. Van Den Bosch E. van Dijken J. P. Scheffers W. A. (1984). Alcoholic Fermentation of D -Xylose by Yeasts. Appl. Environ. Microbiol. 47, 1221–1223. 10.1128/aem.47.6.1221-1223.1984 16346558 PMC240200

[B131] Tran Nguyen Hoang P. Ko J. K. Gong G. Um Y. Lee S.-M. (2018). Genomic and Phenotypic Characterization of a Refactored Xylose-Utilizing *Saccharomyces cerevisiae* Strain for Lignocellulosic Biofuel Production. Biotechnol. Biofuels 11, 268. 10.1186/s13068-018-1269-7 30288173 PMC6162923

[B132] Van Petegem F. Collins T. Meuwis M.-A. Gerday C. Feller G. Van Beeumen J. (2002). Crystallization and Preliminary X-ray Analysis of a Xylanase from the psychrophilePseudoalteromonas Haloplanktis. Acta Crystallogr. D Biol. Cryst. 58, 1494–1496. 10.1107/S0907444902011666 12198313

[B133] Velasco J. Oliva B. Mulinari E. J. Quintero L. P. da Silva Lima A. Gonçalves A. L. (2019). Heterologous Expression and Functional Characterization of a GH10 Endoxylanase from Aspergillus fumigatus Var. Niveus with Potential Biotechnological Application. Biotechnol. Rep. 24, e00382. 10.1016/j.btre.2019.e00382 PMC6881608 31799141

[B134] Verduyn C. Postma E. Scheffers W. A. Van Dijken J. P. (1992). Effect of Benzoic Acid on Metabolic Fluxes in Yeasts: a Continuous-Culture Study on the Regulation of Respiration and Alcoholic Fermentation. Yeast 8, 501–517. 10.1002/yea.320080703 1523884

[B135] Verhamme T. Arents J. C. Postma P. W. Crielaard W. Hellingwerf K. J. (2002). Investigation of *In Vivo* Cross-Talk between Key Two-Component Systems of *Escherichia coli* . Microbiology 148 (Pt 1), 69–78. 10.1099/00221287-148-1-69 11782500

[B136] Wang C. Lu X. Gao J. Li X. Zhao J. (2018). Xylo-oligosaccharides Inhibit Enzymatic Hydrolysis by Influencing Enzymatic Activity of Cellulase from Penicillium oxalicum. Energy Fuels 32, 9427–9437. 10.1021/acs.energyfuels.8b01424

[B137] Weimberg R. (1961). Pentose Oxidation by Pseudomonas Fragi. J. Biol. Chem. 236, 629–635. 10.1016/s0021-9258(18)64279-6 13783864

[B138] Whistler R. L. Masak E. Jr. (1955). Enzymatic Hydrolysis of Xylan1. J. Am. Chem. Soc. 77, 1241–1243. 10.1021/JA01610A042

[B139] Wierzbicki M. P. Maloney V. Mizrachi E. Myburg A. A. (2019). Xylan in the Middle: Understanding Xylan Biosynthesis and its Metabolic Dependencies toward Improving wood Fiber for Industrial Processing. Front. Plant Sci. 10, 1–29. 10.3389/fpls.2019.00176 30858858 PMC6397879

[B140] Wong K. K. Tan L. U. Saddler J. N. (1988). Multiplicity of Beta-1,4-Xylanase in Microorganisms: Functions and Applications. Microbiol. Rev. 52, 305–317. 10.1128/mmbr.52.3.305-317.198810.1128/mr.52.3.305-317.1988 3141761 PMC373146

[B141] Woodward J. Wiseman A. (1982). Fungal and Other β-d-glucosidases - Their Properties and Applications. Enzyme Microb. Tech. 4, 73–79. 10.1016/0141-0229(82)90084-9

[B142] Xia P.-F. Zhang G.-C. Liu J.-J. Kwak S. Tsai C.-S. Kong I. I. (2016). GroE Chaperonins Assisted Functional Expression of Bacterial Enzymes in *Saccharomyces Cerevisiae* . Biotechnol. Bioeng. 113, 2149–2155. 10.1002/bit.25980 27003667

[B143] Xiao W. Li H. Xia W. Yang Y. Hu P. Zhou S. (2019). Co-expression of Cellulase and Xylanase Genes in *Sacchromyces cerevisiae* toward Enhanced Bioethanol Production from Corn stover. Bioengineered 10, 513–521. 10.1080/21655979.2019.1682213 31661645 PMC6844370

[B144] Yegin S. (2017). Single-step Purification and Characterization of an Extreme Halophilic, Ethanol Tolerant and Acidophilic Xylanase from Aureobasidium Pullulans NRRL Y-2311-1 with Application Potential in the Food Industry. Food Chem. 221, 67–75. 10.1016/j.foodchem.2016.10.003 27979257

[B145] Zhang C. Acosta-Sampson L. Yu V. Y. Cate J. H. D. (2017a). Screening of Transporters to Improve Xylodextrin Utilization in the Yeast *Saccharomyces cerevisiae* . PLoS One 12, e0184730. 10.1371/journal.pone.0184730 28886200 PMC5591001

[B146] Zhang G.-C. Turner T. L. Jin Y.-S. (2017b). Enhanced Xylose Fermentation by Engineered Yeast Expressing NADH Oxidase through High Cell Density Inoculums. J. Ind. Microbiol. Biotechnol. 44, 387–395. 10.1007/s10295-016-1899-3 28070721

[B147] Zhang G. M. Huang J. Huang G. R. Ma L. X. Zhang X. E. (2007). Molecular Cloning and Heterologous Expression of a New Xylanase Gene from Plectosphaerella Cucumerina. Appl. Microbiol. Biotechnol. 74, 339–346. 10.1007/s00253-006-0648-3 17115208

[B148] Zhang J. Tang M. Viikari L. (2012). Xylans Inhibit Enzymatic Hydrolysis of Lignocellulosic Materials by Cellulases. Bioresour. Tech. 121, 8–12. 10.1016/j.biortech.2012.07.010 22858461

